# Organic Ammonium Halide Modulators as Effective Strategy for Enhanced Perovskite Photovoltaic Performance

**DOI:** 10.1002/advs.202004593

**Published:** 2021-03-10

**Authors:** Seckin Akin, Bitao Dong, Lukas Pfeifer, Yuhang Liu, Michael Graetzel, Anders Hagfeldt

**Affiliations:** ^1^ Department of Metallurgical and Materials Engineering Karamanoglu Mehmetbey University Karaman Turkey; ^2^ Laboratory of Photomolecular Science École Polytechnique Fédérale de Lausanne Station 6 Lausanne CH‐1015 Switzerland; ^3^ Laboratory of Photonics and Interfaces Department of Chemistry and Chemical Engineering École Polytechnique Fédérale de Lausanne Lausanne CH‐1015 Switzerland

**Keywords:** defect passivation, moisture resistant, perovskite solar cells, surface treatment

## Abstract

Despite rapid improvements in efficiency, long‐term stability remains a challenge limiting the future up‐scaling of perovskite solar cells (PSCs). Although several approaches have been developed to improve the stability of PSCs, applying ammonium passivation materials in bilayer configuration PSCs has drawn intensive research interest due to the potential of simultaneously improving long‐term stability and boosting power conversion efficiency (PCE). This review focuses on the recent advances of improving n‐*i*‐p PSCs photovoltaic performance by employing ammonium halide‐based molecular modulators. The first section briefly summarizes the challenges of perovskite materials by introducing the degradation mechanisms associated with the hygroscopic nature and ion migration issues. Then, recent reports regarding the roles of overlayers formed from ammonium‐based passivation agents are discussed on the basis of ligand and halide effects. This includes both the formation of 2D perovskite films as well as purely organic passivating layers. Finally, the last section provides future perspectives on the use of organic ammonium halides within bilayer‐architecture PSCs to improve the photovoltaic performances. Overall, this review provides a roadmap on current demands and future research directions of molecular modulators to address the critical limitations of PSCs, to mitigate the major barriers on the pathway toward future up‐scaling applications.

## Graphical abstract

Passivating the defects and preventing the degradation of metal halide perovskites by humidity continues to pose a big challenge to the vast scientific community developing this promising new light harvesting material. In this review, recent advances and prospects employing organic ammonium halide‐based passivation materials that attempt to suppress such issues in n–*i*–p architecture perovskite solar cells are discussed.

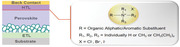

## 1 Introduction

Perovskite solar cells (PSCs) are considered to be the most promising emerging third‐generation photovoltaic technology and their in‐depth investigation is of utmost importance with regard to basic theoretical concepts, experimental observations, and future technology development.^[^ ^1^, ^2^, ^3^ ^]^ Feasible manufacturing and low‐cost fabrication made PSCs an important breakthrough among the different photovoltaic technologies. In general, a typical PSC consists of the perovskite active layer sandwiched between two selective charge transporting materials, the so‐called electron‐transporting layer (ETL) and hole‐transporting layer (HTL). We can distinguish between two main types of device architectures, regular (n–*i*–p) and inverted (p–*i*–n), depending on the stacking order of these components. Of these, n–*i*–p structured PSCs are more commonly studied for the fabrication of stable high efficiency devices.^[^ ^4^, ^5^ ^]^ The power conversion efficiency (PCE) of PSCs recently reached over 25% as a result of careful device engineering and designing of interfaces.^[^ ^6^, ^7^, ^8^, ^9^, ^10^ ^]^ This rapid progress in efficiency highlights how PSCs have benefitted from the use of highly optimized layers and connecting interfaces.

Although having been greatly advanced in terms of PCE, perovskite photovoltaic technology still requires overcoming of the drawbacks associated with defect‐based nonradiative recombination losses on the surface and/or at grain boundaries of perovskite materials in order to be fit for large‐scale deployment.^[^ ^11^, ^12^, ^13^, ^14^ ^]^ Such defects are considered to be related to the low formation energy and/or low thermal stability of perovskite materials containing volatile organic components which can easily evaporate during the annealing process. These defects mostly create shallow electronic levels near the band edges^[^ ^15^, ^16^ ^]^ and have profound, undesired effects on charge carrier dynamics due to a non‐stoichiometric balance of charges.^[^ ^17^, ^18^ ^]^ The formation of defects either at the surface or at grain boundaries of perovskite layers plays a major role in the chemical degradation of perovskite absorbers.^[^ ^19^, ^20^ ^]^ Under operating conditions, these defects can encourage ion migration and permeation of moisture and/or oxygen through the perovskite film and thus reduce the photovoltaic performance of devices.^[^ ^21^ ^]^ It is, therefore, highly desirable to passivate these defects to further improve the PCE and prolong the stability of perovskite devices.^[^ ^22^, ^23^, ^24^ ^]^ In contrast to covalently bonded silicon (Si) solar cells, perovskite materials have a strong ionic character leading to the formation of either positively or negatively charged defects, which needs to be taken into consideration for defect passivation.^[^ ^25^, ^26^ ^]^

Apart from defect‐related instability issues of PSCs, moisture or oxygen uptake are also considered to be important degradation mechanisms.^[^ ^27^ ^]^ Owing to its crucial location in a regular n–*i*–p PSC, the HTL plays a key role in preventing environmental degradation caused by the contact with moisture and/or oxygen.^[^ ^28^ ^]^ In particular, an intrinsically hydrophobic HTL can partially solve these above‐mentioned issues due to the formation of a barrier between the highly hydrophilic ionic perovskite and the surrounding environment. However, the poor compatibility of these two layers and resulting defects at the perovskite/HTL interface have, until now, stood in the way of approaching theoretical limits. The design of 2D perovskites can efficiently improve the stability of perovskites in ambient air by healing the defects located at the perovskite/HTL interface. Besides, novel approaches on the interfacial modification and the employment of inorganic HTL materials or HTL‐free architecture would considerably enhance the stability of ensuing PSCs.^[^ ^21^, ^29^ ^]^ On the other hand, state‐of‐the‐art HTLs such as *N^2^*,*N^2^*,*N^2′^*,*N^2′^*,*N^7^*,*N^7^*,*N^7′^*,*N^7′^*‐octakis(4‐methoxyphenyl)‐9,9′‐spirobi[9*H*‐Fluoren]‐2,2′,7,7′‐tetramin (spiro‐OMeTAD) and poly‐[bis‐(4‐phenyl)‐(2,4,6‐trimethylphenyl)‐amin] (PTAA) exhibit hole mobilities as well as conductivities which are too low for achieving an efficient charge transfer.^[^ ^30^ ^]^ Therefore, the use of dopants/additives such as lithium bis(trifluoromethanesulfonyl)imide (LiTFSI) and *tert*‐butyl pyridine (*t*BP) is inevitable in order to reach acceptable device performances.^[^ ^31^ ^]^ However, in the presence of these additives, the HTL layers become increasingly hydrophilic, thereby diminishing their capacity to act as a protective coating.^[^ ^32^ ^]^ Furthermore, ion migration into and within the perovskite active layer has been found to cause irreversible degradation.^[^ ^33^, ^34^, ^35^ ^]^ In addition, such ionic dopants, specifically Li^+^ ions, are mobile species under operating conditions, causing serious hysteresis and degrading device performance.^[^ ^34^, ^36^, ^37^ ^]^

**Figure 1:**
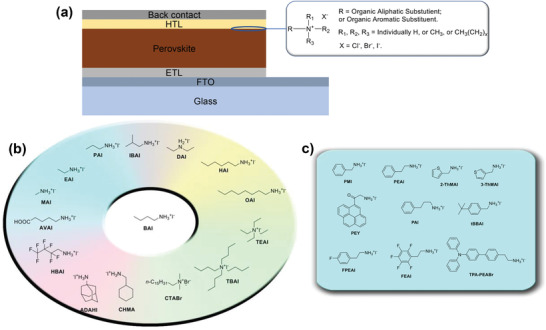
a) The typical device structure of PSCs in n–*i*–p architecture showing general formula for an organic ammonium‐based passivation agent. The molecular structures of some of the b) aliphatic and c) aromatic ammonium halides used in perovskite surface treatment.

Interface engineering is a facile and effective way to eliminate both intrinsic and environmental degradation mechanisms, simultaneously. Various approaches based on this principle have so far been developed and employed in PSCs.^[^ ^38^, ^39^, ^40^, ^41^, ^42^ ^]^ Especially ammonium halides have demonstrated a remarkable potential owing to their tunable electro‐chemical and optical properties, for instance, their interaction with lead cations, hydrophobicity and optical behavior, as well as low production costs and solution processability.^[^ ^26^, ^43^, ^44^ ^]^ A general formula for an organic ammonium‐based passivation agent can be abbreviated as R—N^1^R^2^R^3^R^+^X^−^ (**Figure** **1**a), where R and X represent an organic moiety and monovalent halide anion, respectively. ^1^R, ^2^R_,_ and ^3^R are supplementary groups attached to the central nitrogen atom, and are, in most instances, small moieties like H—, CH_3_— or CH_3_CH_2_—. Organic ammonium halides with an ammonium functional group (—NR_3_^+^) exhibit an efficient role as passivation agents. In contrast to amines (—NH_2_) which are capable of coordinating to positively charged Lewis acid defects due to their electron lone pair, ammonium cations mostly heal negatively charged defects through electrostatic interactions including ionic bonding and hydrogen bonding.^[^ ^45^ ^]^ Ammonium halides normally exhibit strong interactions with the ionic perovskite materials in general and charged surface defects in particular, thereby mitigating losses from charge carrier recombination. Hitherto, different kinds of ammonium halides have played an important role in this defect‐healing process to impede charge carrier recombination losses with their negatively and/or positively charged components. The formation of a thin (quasi‐)2D perovskite layer (A_2_PbX_4_)*_x_*(FAPbI_3_)*_y_* (A: organic ligand, FA: formamidinium) upon surface treatment of the bulk perovskite material with an organic ammonium halide is frequently reported, for which some studies have either indicated a consumption of excess PbI_2_ or perovskite.^[^ ^8^ ^]^ Others are generally referring to the formation of a thin layer consisting of pristine passivation material without formation of a low‐dimensional perovskite layer. Although having been widely studied, a general rationale for formation of a 2D layer by reacting with the perovskite versus simple surface passivation remains unrevealed, but it is believed to be highly dependent on the properties of the passivator and the perovskite active layer as well as the processing method. Several criteria for forming a 2D layer on the bulk's surface are proposed below: i) the ligand has to fit within a certain size tolerance to be located into the A site of A_2_PbX_4_ based perovskites, ii) primary ammonium cations are preferred to fit into the vacancies, and to coordinate with the exposed [PbI_6_] octahedra to form 2D crystals and iii) in some cases, elevated temperature is considered to facilitate the formation of a 2D phase.^[^ ^46^ ^]^

The alkyl group(s) (R—) can therefore be tailored to yield the desired properties. Currently, most organic perovskite surface treatment materials are based on either a linear aliphatic ammonium halide or aromatic ammonium halide, with butylammonium iodide (BAI)^[^ ^47^ ^]^ and phenylethylammonium iodide (PEAI)^[^ ^48^ ^]^ as common representatives (Figure 1b,c). Although the aliphatic butyl group BA^+^ is considered to be a spacer, which is optically and electrochemically inert, modifications on the aliphatic chain can also have positive effects on the performance of perovskite devices. The second family of organic passivators contains aromatic moieties, making use of their spacer as well as electronic effect simultaneously. As the simplest derivatives of aromatic ammonium iodide, PEAI and benzylammonium iodide (BAI) are the most widely studied and used aromatic ammonium halide passivation materials.^[^ ^49^ ^]^ Although most corresponding reports are discussing properties other than a potential electronic effect, such as influences related to their hydrophobicity and them acting as spacers, the electronic effects of aromatic passivators have started to draw research attention, due to a potentially more favored hole‐extraction from the perovskite photoabsorber into the HTL.^[^ ^50^ ^]^

In addition to the organic ammonium cations, the halide counterions can also exhibit significant effects on the performance and durability of perovskite devices and can therefore be tailored to yield the desired properties. In most cases, iodide (I^−^), bromide (Br^−^), or chloride (Cl^−^) are used. In particular, Cl^−^ ions in ammonium salts can exchange with halides in the perovskite network and thus retard the crystallization rate to obtain larger grains and better morphologies.^[^ ^51^, ^52^ ^]^ On the other hand, Br^−^ containing ammonium salts have proven to be able to increase the photovoltage by causing band bending within the interfaces.^[^ ^53^ ^]^ It is also known that I^−^ anions can passivate halide vacancies, suppressing a potential pathway for nonradiative recombination.^[^ ^54^, ^55^ ^]^

In this review, we provide an overview of the recent progress on the strategies adopted to passivate perovskite defects using ammonium halide based agents to achieve high‐efficiency and long‐term stability of PSCs of n–*i*–p architecture. **Table** **1** summarizes the recent progress on the photovoltaic performance of organic ammonium halide modulator‐based PSCs. First, we aim to highlight the ammonium halide‐based approaches that have been reported to heal the defect states at the perovskite/HTL interface. The next section defines the different types of organic backbones used for the preparation of ammonium ligands and summarizes the effects of these ligands on the photovoltaic performance and durability of PSCs. The effects of counterions regarding defect healing and photovoltaic performance are discussed in the following section. Finally, the last section provides some future perspective on defect passivation in PSCs.

**Table 1:** Summary of the photovoltaic performance of n‐*i*‐p PSCs employing ammonium halide‐based molecular modulators

Passivation agent	Device configuration	*V*oc [V]	*J*sc [mA cm^−2^]	FF [%]	PCE [%]	Ref.
*N*‐butylammonium iodide	FTO/c‐TiO_2_/MAPbI_3_/spiro‐OMeTAD/Au	1.08	16.56	62.0	11.5	^[^ ^47^ ^]^
*N*‐butylammonium iodide	FTO/c‐TiO_2_/mp‐TiO_2_/Cs_0.05_(MA_0.17_FA_0.83_)Pb(I_0.83_Br_0.17_)_3_/spiro‐OMeTAD/Au	1.06	19.40	76.9	15.7	^[^ ^55^ ^]^
*N*‐propylammonium iodide	FTO/c‐TiO_2_/mp‐TiO_2_/FA_0.79_MA_0.16_Cs_0.05_PbI_2.5_Br_0.5_/spiro‐OMeTAD/Au	1.06	21.90	74.4	17.2	^[^ ^56^ ^]^
Ethylammonium iodide	FTO/c‐TiO_2_/mp‐TiO_2_/FA_0.9_Cs_0.07_MA_0.03_Pb(I_0.92_Br_0.08_)_3_/spiro‐OMeTAD/Au	1.12	24.14	81.0	22.4	^[^ ^57^ ^]^
Methylammonium iodide	FTO/c‐TiO_2_/mp‐TiO_2_/MAPbI_3_/spiro‐OMeTAD/Au	—	—	—	17.2	^[^ ^58^ ^]^
*Iso*‐butyl ammonium iodide	FTO/c‐TiO_2_/mp‐TiO_2_/(FAPbI_3_)_0.85_(MAPbBr_3_)_0.15_/spiro‐OMeTAD/Au	—	—	—	21.7	^[^ ^59^ ^]^
*Iso*‐butyl ammonium iodide	FTO/c‐TiO_2_/mp‐TiO_2_/FAPbI_3_/spiro‐OMeTAD/Au	1.11	25.40	80.5	22.7	^[^ ^8^ ^]^
Diethylammonium bromide	ITO/TiO*_x_*/MAPbI_3_/spiro‐OMeTAD/MoO_3_/Ag	1.06	21.93	78.7	18.3	^[^ ^60^ ^]^
Hexylammonium iodide	FTO/SnO_2_/CsFAMAPb(I,Br)_3_/spiro‐OMeTAD/Ag	1.14	23.76	76.0	20.6	^[^ ^61^ ^]^
Octylammonium iodide	FTO/c‐TiO_2_/mp‐TiO_2_/Cs_0.05_(MA_0.17_FA_0.83_)Pb(I_0.83_Br_0.17_)_3_/spiro‐OMeTAD/Au	1.02	19.37	76.7	15.2	^[^ ^55^ ^]^
Tetra‐ethyl ammonium iodide	FTO/c‐TiO_2_/MAPbI_3_/spiro‐OMeTAD/Ag	0.99	19.60	67.0	12.9	^[^ ^62^ ^]^
Cetyl trimethyl ammonium bromide	ITO/SnO_2_:CNDs/MAPbI_3_/spiro‐OMeTAD/Au	1.11	23.20	74.0	18.9	^[^ ^63^ ^]^
5‐Ammoniumvaleric acid iodide	FTO/c‐TiO_2_/mp‐TiO_2_/MAPbI_3_/spiro‐OMeTAD/Au	1.06	22.30	76.0	18.0	^[^ ^64^ ^]^
5‐Ammoniumvaleric acid iodide	FTO/c‐TiO_2_/SnO_2_/MAPbI_3_/spiro‐OMeTAD/Ag	1.11	22.92	79.0	20.1	^[^ ^65^ ^]^
CF_3_(CF_2_)_2_CH_2_NH_3_I:formamidinium bromide	FTO/c‐TiO_2_/mp‐TiO_2_/Cs_0.05_MA_0.1_FA_0.85_PbI_2.9_Br_0.1_/spiro‐OMeTAD/Au	1.15	25.26	79.0	22.8	^[^ ^66^ ^]^
Adamantane	FTO/c‐TiO_2_/mp‐TiO_2_/CsFAMAPb(I,Br)_3_/spiro‐OMeTAD/Au	1.14	22.53	79.3	20.5	^[^ ^67^ ^]^
1‐Adamantylamine	FTO/c‐TiO_2_/mp‐TiO_2_/CsFAMAPb(I,Br)_3_/spiro‐OMeTAD/Au	1.16	22.57	80.4	20.9	^[^ ^67^ ^]^
Protonated 1‐adamantylammonium iodide	FTO/c‐TiO_2_/mp‐TiO_2_/CsFAMAPb(I,Br)_3_/spiro‐OMeTAD/Au	1.16	24.30	77.3	21.9	^[^ ^68^ ^]^
Cyclohexylammonium iodide	FTO/c‐TiO_2_/mp‐TiO_2_/MAPbI_3_/spiro‐OMeTAD/Au	0.86	21.60	61.8	11.5	^[^ ^69^ ^]^
Cyclohexylammonium iodide	FTO/c‐TiO_2_/mp‐TiO_2_/MA_1‐_ *_x_*Cs*_x_*PbI_3_/spiro‐OMeTAD/Au	0.90	23.60	58.9	12.6	^[^ ^69^ ^]^
Phenylethylammonium iodide	FTO/c‐TiO_2_/mp‐TiO_2_/Cs_0.1_FA_0.74_MA_0.13_PbI_2.48_Br_0.39_/spiro‐OMeTAD/Au	1.15	22.73	79.4	20.8	^[^ ^70^ ^]^
Phenethylammonium iodide	ITO/SnO_2_/FA_1−_ *_x_*MA*_x_*PbI_3_/spiro‐OMeTAD/Au	1.18	25.20	78.4	23.3	^[^ ^46^ ^]^
Phenethylammonium iodide	FTO/c‐TiO_2_/mp‐TiO_2_/Cs_0.05_(FA_0.83_MA_0.17_)_0.95_Pb(I_0.83_Br_0.17_)_3_/spiro‐OMeTAD/Au	1.11	22.89	73.0	18.5	^[^ ^71^ ^]^
Phenethylammonium iodide	FTO/c‐TiO_2_/MAPbI_3_/spiro‐OMeTAD/Au	1.11	20.53	75.0	16.8	^[^ ^47^ ^]^
Phenethylammonium iodide	FTO/c‐TiO_2_/FAPbI_3_/spiro‐OMeTAD/Au	1.09	23.76	76.8	19.8	^[^ ^73^ ^]^
4‐Fluorophenethylamin iodide	FTO/c‐TiO_2_/FAPbI_3_/spiro‐OMeTAD/Au	1.06	22.13	72.8	17.1	^[^ ^73^ ^]^
Phenethylammonium iodide:formamidinium iodide (1:1)	FTO/c‐TiO_2_/FAPbI_3_/spiro‐OMeTAD/Au	1.14	24.20	76.6	21.2	^[^ ^73^ ^]^
4‐Trifluoromethyl phenethylammonium	ITO/ZnO/CsPbI_2_Br/CFPEAI/spiro‐OMeTAD/MoO_3_/Ag	1.23	15.45	84.7	16.1	^[^ ^86^ ^]^
4‐Fluorophenylethylammonium iodide	FTO/c‐TiO_2_/mp‐TiO_2_/FAPbI_3_/spiro‐OMeTAD/Au	0.95	24.10	67.7	15.5	^[^ ^74^ ^]^
1‐(Ammonium acetyl)pyrene	FTO/SnO_2_/(PEY_2_PbI_4_)_0.02_MAPbI_3_/spiro‐OMeTAD/Au	1.05	21.15	66.1	14.7	^[^ ^75^ ^]^
2‐Thiophenemethylammonium iodide	FTO/c‐TiO_2_/mp‐TiO_2_/p‐SnO_2_/[(FAPbI_3_)_0.87_(MAPbBr_3_)_0.13_]_0.92_(CsPbI_3_)_0.08_/spiro‐OMeTAD/Au	1.13	23.50	75.1	19.9	^[^ ^76^ ^]^
3‐Thiophenemethylammonium iodide	FTO/c‐TiO_2_/mp‐TiO_2_/p‐SnO_2_/[(FAPbI_3_)_0.87_(MAPbBr_3_)_0.13_]_0.92_(CsPbI_3_)_0.08_/spiro‐OMeTAD/Au	1.13	23.60	77.1	20.6	^[^ ^76^ ^]^
2‐Thiopheneethylammonium iodide	FTO/c‐TiO_2_/mp‐TiO_2_/p‐SnO_2_/[(FAPbI_3_)_0.87_(MAPbBr_3_)_0.13_]_0.92_(CsPbI_3_)_0.08_/spiro‐OMeTAD/Au	1.18	23.60	73.7	19.4	^[^ ^76^ ^]^
2‐Thiophenemethylammonium iodide	FTO/c‐TiO_2_/mp‐TiO_2_/p‐SnO_2_/[(FAPbI_3_)_0.87_(MAPbBr_3_)_0.13_]_0.92_(CsPbI_3_)_0.08_/spiro‐OMeTAD/Au	1.10	23.20	74.0	18.9	^[^ ^78^ ^]^
Phenylethylammonium iodide	FTO/c‐TiO_2_/mp‐TiO_2_/p‐SnO_2_/[(FAPbI_3_)_0.87_(MAPbBr_3_)_0.13_]_0.92_(CsPbI_3_)_0.08_/spiro‐OMeTAD/Au	1.10	23.40	74.0	19.1	^[^ ^78^ ^]^
Aniline	FTO/c‐TiO_2_/FAPbI_3_/spiro‐OMeTAD/Au	0.93	23.00	64.0	13.8	^[^ ^49^ ^]^
Benzylamine	FTO/c‐TiO_2_/FAPbI_3_/spiro‐OMeTAD/Au	1.12	23.60	73.0	19.2	^[^ ^49^ ^]^
Phenethylamine	FTO/c‐TiO_2_/FAPbI_3_/spiro‐OMeTAD/Au	0.95	23.60	59.0	13.3	^[^ ^49^ ^]^
Phenylethylammonium iodide	FTO/c‐TiO_2_/mp‐TiO_2_/Cs0_.05_FA_0.85_MA_0.10_Pb(I_0.97_Br_0.03_)_3_/spiro‐OMeTAD/Au	1.12	25.01	80.9	22.7	^[^ ^79^ ^]^
4‐*tert*‐butyl‐benzylammonium iodide	FTO/c‐TiO_2_/mp‐TiO_2_/Cs0_.05_FA_0.85_MA_0.10_Pb(I_0.97_Br_0.03_)_3_/spiro‐OMeTAD/Au	1.14	25.10	82.1	23.5	^[^ ^79^ ^]^
2‐(4‐Fluorophenyl)ethyl ammonium iodide	FTO/c‐TiO_2_/mp‐TiO_2_/Cs_0.1_(FA_0.83_MA_0.17_)_0.9_Pb(I_0.83_Br_0.17_)_3_/spiro‐OMeTAD/Au	1.13	22.80	80.0	20.5	^[^ ^80^ ^]^
2‐(2‐Fluorophenyl)ethylamine iodide	FTO/c‐SnO_2_/Cs_0.05_FA_0.79_MA_0.16_PbI_2.49_Br_0.51_/spiro‐OMeTAD/Au	1.17	22.62	77.9	20.6	^[^ ^82^ ^]^
2‐(3‐Fluorophenyl)ethylamine iodide	FTO/c‐SnO_2_/Cs_0.05_FA_0.79_MA_0.16_PbI_2.49_Br_0.51_/spiro‐OMeTAD/Au	1.16	22.75	76.8	20.5	^[^ ^82^ ^]^
2‐(4‐Fluorophenyl)ethylamine iodide	FTO/c‐SnO_2_/Cs_0.05_FA_0.79_MA_0.16_PbI_2.49_Br_0.51_/spiro‐OMeTAD/Au	1.15	22.23	79.5	20.4	^[^ ^82^ ^]^
Phenylammonium iodide	FTO/c‐TiO_2_/mp‐TiO_2_/FA_0.9_Cs_0.1_PbI_2.9_Br_0.1_/spiro‐OMeTAD/Au	0.98	23.18	72.6	16.5	^[^ ^83^ ^]^
Phenylmethylammonium iodide	FTO/c‐TiO_2_/mp‐TiO_2_/FA_0.9_Cs_0.1_PbI_2.9_Br_0.1_/spiro‐OMeTAD/Au	1.02	22.85	74.2	17.3	^[^ ^83^ ^]^
Phenylethylammonium iodide	FTO/c‐TiO_2_/mp‐TiO_2_/FA_0.9_Cs_0.1_PbI_2.9_Br_0.1_/spiro‐OMeTAD/Au	1.04	23.16	75.5	18.1	^[^ ^83^ ^]^
Pentafluoro‐phenylethylammonium iodide	FTO/c‐TiO_2_/mp‐TiO_2_/perovskite/spiro‐OMeTAD/Au	1.10	25.80	78.4	22.2	^[^ ^84^ ^]^
*N*‐((4‐(*N*,*N*,*N*‐triphenyl)phenyl)‐ethyl)ammonium bromide	ITO/SnO_2_:Li/(FAPbI_3_)_0.97_(MAPbBr_3_)_0.03_/spiro‐OMeTAD/Au	1.09	23.13	72.0	18.2	^[^ ^50^ ^]^

## 2 Organic Substituent Effect

As mentioned in the previous chapter, the organic substituent on the ammonium cation can be tailored to achieve the desired, such as spatial, optoelectronic, and hydrophobic effects. Although spatial effects exist for all variations of organic substituents, including aliphatic and aromatic, detailed systematic studies normally make use of aliphatic compounds, which are easier to be adjusted systematically via tuning the hydrocarbon alkyl chain size. On the other hand, optoelectronic effects are only observed in aromatic substituents. In addition, the more accessible chemical reactivity of aromatic hydrocarbons makes them easier to be functionalized to achieve the desired properties, for example, by increasing their hydrophobicity. As a result, to present a clear vision on the effects of organic substituents, the following discussion will be divided into aliphatic and aromatic moieties, with the former emphasizing the observed spatial effects, and the latter focusing on other properties.

### 2.1 Linear or Branched Aliphatic Organic Substituents

**Figure 2:**
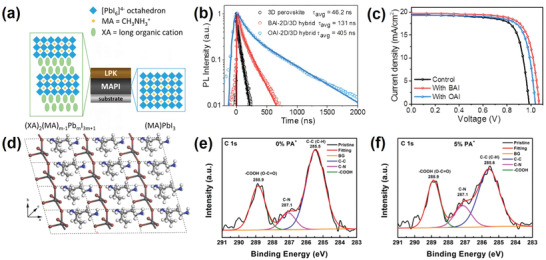
a) Schematic illustration of the crystal structures of methylammonium lead iodide and a layered perovskite (LPK), forming the junction. Reproduced with permission.^[^ ^47^ ^]^ Copyright 2020, American Chemical Society. b) PL decay measurement of different perovskite films (10 mm BAI/OAI for 3D/2D perovskites). Reproduced with permission.^[^ ^55^ ^]^ Copyright 2020, Royal Society of Chemistry. c) *J–V* characteristics of solar cells fabricated from various perovskite layers (10 mm BAI/OAI for 3D/2D perovskites), recorded in the reverse scanning direction with a sweeping rate of 100 mV s^−1^. Reproduced with permission.^[^ ^55^ ^]^ Copyright 2020, Royal Society of Chemistry. d) Top view of the optimized structure of PA_2_PbI_4_. White: H, blue: N, light gray: C, dark gray: Pb, red: I. The lattice parameters for the unit cell of PA_2_PbI_4_ are *a* = 11.616 Å; *b* = 6.150 Å; *c* = 7.514 Å; *α* = 105.557; *β* = 110.664°; and *γ* = 97.809°. Reproduced with permission.^[^ ^56^ ^]^ Copyright 2020, American Chemical Society. High‐resolution XPS for the C 1s peak in (PA)_2_ *_x_*FA_0.79_MA_0.16_Cs_0.05_Pb_1+_ *_x_*I_2.5+4_ *_x_*Br_0.5_‐mixed perovskite films with e) 0% and f) 5% PA^+^ additive. Reproduced with permission.^[^ ^56^ ^]^ Copyright 2020, American Chemical Society.

As a well‐known cation of ammonium halide molecular passivators, *n*‐butylammonium (BA^+^) is one of the most widely used spacer cations for forming 2D perovskites to mitigate defect states. BAI was found to be able to form a thin 2D layer on perovskite surfaces via a solution‐based process. Apart from effective surface passivation, the in situ generated, thin 2D layer based on BA^+^ also functions as an electron‐blocking layer as well as a humidity barrier, effectively suppressing charge recombination and increasing stability against ambient moisture. Docampo et al. were the first to use BA^+^ cation to fabricate perovskite/perovskite heterojunction devices.^[^ ^47^ ^]^ They developed a facile solution‐based cation infiltration process to deposit layered perovskite structures onto 3D methylammonium lead iodide (MAPbI_3_) films combining the advantages of both components (**Figure** **2**a). The 3D MAPbI_3_ perovskite ensures efficient light absorption and charge generation, whereas the top 2D film serves as a selective charge extraction layer and moisture barrier. Since the butyl moiety is too large to be incorporated into the 3D perovskite matrix, it can only form a layered compound on top of the 3D perovskite layer. The obtained results indicated that this layered perovskite structure may act as a selective hole extraction layer for 3D perovskite, leading to reduced recombination losses within the device. Despite the possible barrier effect of the organic substituents, a considerable enhancement in *V*_OC_ (≈80 mV) was reported after optimization of film thickness.

In a related investigation, BAI‐based alkylammonium halide perovskites were formed on a Cs_0.05_(MA_0.17_FA_0.83_)Pb(I_0.83_Br_0.17_)_3_ perovskite film to passivate interfacial defects and retard moisture induced degradation.^[^ ^55^ ^]^ The hybrid 3D/2D perovskite films exhibited longer photoluminescence lifetimes indicating passivation of cationic and halide vacancies on the surface and/or grain boundaries (Figure 2b). As a result, the PCE of the studied perovskite devices improved by 11% compared to typical 3D perovskite‐based devices, reaching 15.94% (Figure 2c). More importantly, the 2D/3D stacked bilayer structure showed a much‐improved stability, maintaining 86% of its initial PCE in comparison to only 61% for its 3D counterpart when subjected to moisture stress under ≈70% relative humidity at ambient temperature.

BAI is most commonly studied due to its wide availability. Because it proved to be unable to incorporate into the perovskite lattice due to its size, researchers are interested in smaller‐sized derivatives of BAI to verify their properties in perovskite. Accordingly, *n*‐propylammonium iodide (PAI) and ethylammonium iodide (EAI) were synthesized and their effects on PSCs verified. PAI was reported by Yao et al. to be able to improve the stability of the FA_0.79_MA_0.16_Cs_0.05_PbI_2.5_Br_0.5_ perovskite film through surface treatment.^[^ ^56^ ^]^ The formation of 2D (PA)_2_PbI_4_ perovskite as a result of the reaction between PAI and excess PbI_2_ was supported by density functional theory calculations and XRD patterns (Figure 2d). Accordingly, PA^+^ was confirmed as not being able to incorporate into the perovskite lattice but forming a thin 2D layer on the surface of the perovskite photoabsorber instead. X‐ray photoelectron spectroscopy (XPS) revealed fewer −COOH (carboxyl) groups on the surface of the passivated perovskite films, indicating a suppressed penetration of oxygen and moisture into the material (Figure 2e,f). This result was further underpinned by surface wettability testing of the (PA)_2_PbI_4_ film revealing a contact angle of >110° confirming excellent hydrophobic properties. Efficiencies as high as 17.23% with 5% PAI additive were achieved. Similar to other studies based on ammonium passivation, the devices’ *V*_OC_ and FF parameters were improved while a slight reduction in *J*_SC_ was explained on the basis of lower light absorption of the PAI‐based perovskite films. After aging in an ambient environment for 2000 h, the device containing 5% PAI demonstrated much better stability with nearly 50% of PCE being retained whereas the efficiency of a pristine control cell dropped below 4%.

**Figure 3:**
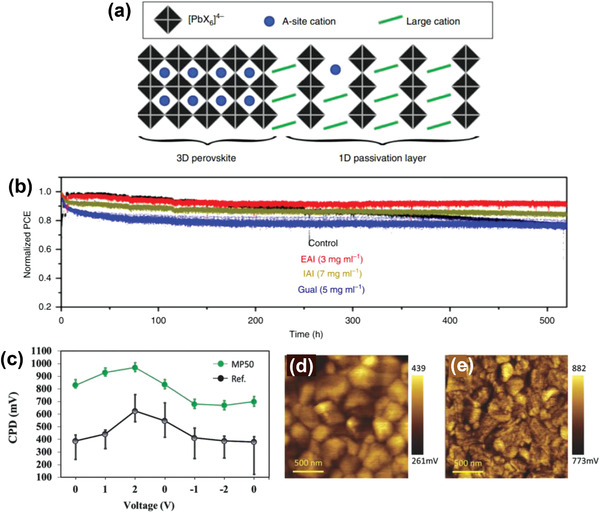
a) Schematic representation of the 1D/3D heterostructure evidenced by solid‐state NMR proximity measurements. b) A comparison of operational stability of control and treated perovskite devices. The devices were measured under a nitrogen environment at room temperature under constant illumination (LED source, ≈1 Sun) at a maximum power point for 550 h. a,b) Reproduced with permission.^[^ ^57^ ^]^ Copyright 2020, Springer Nature. c) Average CPD values as a function of bias voltage applied to the tip for the reference and MP50 treatment samples measured in dark. Spatial CPD maps of the d) reference and e) MP50‐treated samples. c–e) Reproduced with permission.^[^ ^59^ ^]^ Copyright 2020, Wiley‐VCH.

Ethylammonium iodide (EAI), as en example of a ligand with further decreased substituent size, was also capable of reducing the electronic defects at the perovskite/HTL interface.^[^ ^57^ ^]^ EAI was used to modify triple‐cation FA_0.9_Cs_0.07_MA_0.03_Pb(I_0.92_Br_0.08_)_3_ perovskite surface which showed an improvement in *V*_OC_ by 40 mV, leading to PCEs as high as 22.4%. The increase in PL intensity of EAI treated perovskite films further confirmed a reduction of nonradiative recombination losses that could be explained by defect passivation induced by cation exchange and filling of iodide vacancies at the absorber surface. 2D‐solid‐state‐NMR analysis was applied to unravel the atomic level mechanisms of this passivation effect as being linked to the formation of a 27 nm thick 1D EAPbI_3_ passivation layer (**Figure** **3**a). The operational stability of PSCs under working conditions was also monitored. The best performing device showed a loss in efficiency of only 5% under full sunlight intensity with maximum power tracking for 550 h (Figure 3b).

Similarly, the effect of methylammonium cation (MA^+^) on perovskite surfaces is potentially interesting. Hawash et al. reported a facile strategy to tailor the interface between the MAPbI_3_ perovskite and the HTL by vacuum deposition of an ultrathin layer of excess methylammonium iodide (MAI).^[^ ^58^ ^]^ Nominal thicknesses measured with a calibrated quartz crystal microbalance (QCM) of the evaporated ultrathin MAI films were 1, 2, 4, 8, 16, and 32 nm. The fabricated PSCs with optimal MAI film thickness (4 nm) exhibited an average steady‐state PCE of 17.2 ± 0.4%, which was ≈20% higher than that of the reference cells (14.5 ± 1.9%). In addition, device steady‐state PCE revealed a smaller standard deviation of 0.4% as compared to 2% for reference cells, suggesting a significant improvement of device reproducibility.

*Iso*‐butyl ammonium iodide (IBAI) was explored and proved to be effective for improving the photovoltaic performance of PSCs as a structural isomer of BAI. Cho et al. introduced a mixed passivation treatment by employing a combination of formamidinium iodide (FAI) and IBAI to passivate the interface between (FAPbI_3_)_0.85_(MAPbBr_3_)_0.15_ and spiro‐OMeTAD HTL.^[^ ^59^ ^]^ It was found that the role of IBAI is beneficial not only by assisting the formation of a 2D perovskite surface passivation layer during the reaction with excess PbI_2_ but also by controlling the morphology of the 3D film. A significant reduction of the photocurrent hysteresis associated with the formation of an interfacial energetic barrier was demonstrated by Kelvin probe force microscopy (KPFM) showing higher contact potential difference (CPD) (Figure 3c–e). Owing to increased *V*_OC_ and FF values, the best performing device achieved a PCE of 21.7% when using a 1:1 (IBAI to FAI) molar ratio. The improvements in the photovoltaic parameters were further ascribed to passivation effects and a modified energy band alignment at the perovskite/HTL interface. A notable improvement in device stability regarding exposure to moisture was achieved owing to the hydrophobic nature of the *iso‐*butyl ammonium cation, maintaining over 87% of the initial efficiency after 38 days’ storage in an ambient environment featuring 75 ± 20% relative humidity.

**Figure 4:**
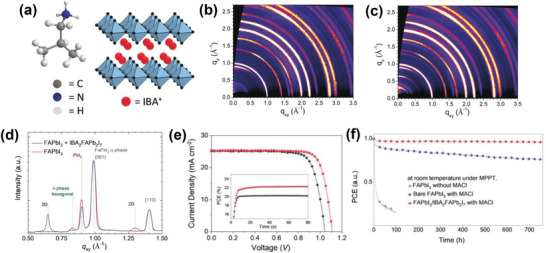
a) Chemical structure of IBA^+^ and schematic illustration of pure 2D‐IBA_2_PbI_4_ perovskite. GIWAXS data of b) FAPbI_3_ film c) FAPbI_3_/2D IBA_2_FAPb_2_I_7_. Angle of incidence was 0.148. d) Radially integrated GIWAXS data from (b,c). e) *J–V* curves of the best‐performing devices. The inset shows the MPP tracking data of PSCs based on bare FAPbI_3_ and FAPbI_3_/IBA_2_FAPb_2_I_7_ perovskites. f) Ageing results of PSCs based on bare FAPbI_3_ (black or blue dotted line) FAPbI_3_/IBA_2_FAPb_2_I_7_ perovskites (red dotted line). Reproduced with permission.^[^ ^8^ ^]^ Copyright 2020, Wiley‐VCH.

Very recently, our group showed the stabilization of *α*‐FAPbI_3_ phase by covering it with 2D IBA_2_FAPb_2_I_7_ (*n* = 2) perovskite photoabsorber as a spacer layer (**Figure** **4**a).^[^ ^8^ ^]^ The obtained stacked 2D/3D perovskite films exhibited long‐lived charge carriers with lifetimes exceeding 1.5 µs. Grazing incidence wide‐angle X‐ray scattering (GIWAXS) analysis was performed to investigate the structure of the overlayer formed by IBAI surface treatment. This revealed distinct diffraction patterns indexed to the structure of the 2D‐perovskite (Figure 4b,c). A unique advantage of IBAI is that surface treatment of FAPbI_3_ perovskite with IBAI spontaneously gives rise to the formation of IBA_2_FAPb_2_I_7_ (*n* = 2) phase instead of *n* = 1 IBA_2_PbI_4_ (Figure 4d). Besides offering effective passivation and protection of the underneath 3D perovskite layer, 2D perovskites with larger *n* values are considered to have a better orbital overlap, thus giving rise to smaller barriers for hole‐extraction, as evident from the superior photovoltaic parameters obtained from IBA_2_FAPb_2_I_7_‐containing devices. These features translated into a high photovoltage of 1113 mV compared to 1053 mV for the reference system. This resulted in a PCE close to 23 % (Figure 4e), which was the record for FAPbI_3_‐based PSCs at the time. In addition, the *α*‐FAPbI_3_/IBA_2_FAPb_2_I_7_ based cell showed excellent operational stability (Figure 4f), retaining around 85% of its initial efficiency after being exposed to severe combined heat and light stress. This included simultaneous exposure to full simulated sunlight with maximum power point tracking at 80 °C for 500 h.

**Figure 5:**
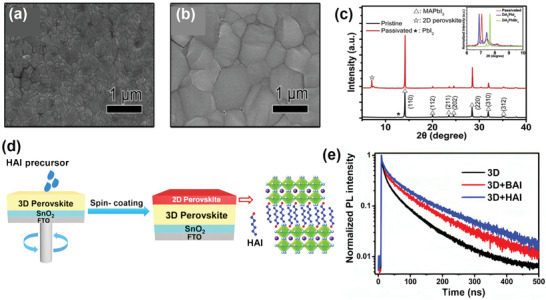
Top view of SEM images of a) pristine film and b) 5 mg mL^−1^ DABr solution treated perovskite films. c) X‐ray diffraction patterns of pristine and DABr‐treated perovskite films. The inset shows that the three diffraction peaks of the passivated perovskite hybrid at 7.06°, 7.45°, and 8.18° mainly arose from DA_2_PbI_4_, and the weak diffraction peak at 7.45° belongs to DA_2_PbBr_4_. a–c) Reproduced with permission.^[^ ^60^ ^]^ Copyright 2020, Elsevier. d) Fabrication process of the 2D perovskite based on HAI in the 2D/3D stacking structure. e) The time‐resolved photoluminescence (TRPL) decay curves of 3D, 3D + BAI, and 3D + HAI perovskite films samples. d,e) Reproduced with permission.^[^ ^61^ ^]^ Copyright 2020, American Chemical Society.

A secondary ammonium isomer of BA^+^, diethylammonium (DA^+^) was investigated in planar PSCs for post‐treatment of MAPbI_3_ perovskite films.^[^ ^60^ ^]^ As a result of the direct reaction between diethylammonium bromide (DABr) and excess PbI_2_ in the MAPbI_3_ films, highly uniform DA_2_PbI*_x_*Br_4–_ *_x_* capped MAPbI_3_ hybrid films were formed, exhibiting good morphology with larger grains and fewer pinholes (**Figure** **5**a,b). The formation of DA_2_PbI*_x_*Br_4−_ *_x_* capping layer was confirmed by XRD patterns exhibiting three diffraction peaks at low angles (Figure 5c). The slight blue shift of the absorption edge of the 2D/3D perovskite film was attributed to the incorporation of Br^−^. Moreover, the DA_2_PbI*_x_*Br_4−_ *_x_* capped MAPbI_3_ surface possesses a well‐matched energy level, with DA_2_PbI*_x_*Br_4‐_ *_x_* acting as an interfacial layer to promote the dissociation of photogenerated carriers and suppress interfacial recombination. Consequently, a PCE of 18.30% was achieved, which was 27% higher than that of the control device (14.37%). Stability tests showed that the PCE of the control device drops below 95% of the initial value upon continuous illumination for 20 h, whereas the passivated device still maintains 95% of the initial value following continuous illumination for 30 h.

Recently, Lv et al. showed that the chain length of organic ammonium iodide is a crucial factor concerning defect passivation.^[^ ^61^ ^]^ Utilizing organic ammonium iodides containing long‐chain organic cations to in situ grow 2D perovskite layers on top of the CsFAMAPb(I,Br)_3_ perovskite film is not only efficient at simultaneously passivating both organic cation (such as MA^+^) and iodide vacancies but also with regard to promoting air stability due to the hydrophobic organic cations (Figure 5d). Compared to BAI, a hexylammonium iodide (HAI)‐derived 2D perovskite was found to be more efficient in decreasing interfacial defects in 2D/3D PSCs. Compared with pure 3D and BAI‐2D/3D films, the HAI‐2D/3D structure exhibited an increased photoluminescence lifetime by effectively suppressing interfacial charge recombination owing to the presence of fewer interfacial defects (Figure 5e). As a consequence, the PCE of HAI‐2D/3D improved to 20.62% as compared to the pure 3D (18.83%) and BAI‐2D/3D (19.43%) counterparts. This improvement was mainly due to an increase in *V*_OC_ (from 1.10 V (3D) and 1.12 V (BAI‐2D/3D) to 1.14 V (HAI‐2D/3D)). Furthermore, the PSCs based on HAI‐2D/3D retained their as‐prepared PCE even after storage for 50 days in an environment with a relative humidity of 55−75% and a temperature of 25−40 °C, while that of 3D and BAI‐2D/3D devices decreased by more than 20%. Furthermore, the device employing pure 3D perovskite degraded to 60% of its original PCE in an oven at 85 °C, while the corresponding BAI‐2D/3D and HAI‐2D/3D still maintained 80% of their initial efficiencies after continuous heating for 600 h. This outstanding thermal stability was explained on the basis of efficient defect passivation induced by the 2D perovskite capping layer. This work once again showed that long‐chain organic ammonium salts are promising for further boosting the PCE and stability of PSCs.

Koh et al. also investigated the effects of octylammonium iodide (OAI) as an organic salt on Cs_0.05_(MA_0.17_FA_0.83_)Pb(I_0.83_Br_0.17_)_3_ perovskite layer.^[^ ^55^ ^]^ Similar to BAI, OAI also formed a thin 2D perovskite layer on the surface of the 3D material after annealing at 100 °C, which acted as a moisture repelling layer (water contact angle >90°) thereby enhancing the stability of the entire film. The reaction of OAI with the bulk perovskite and the existence of a 2D layer was further confirmed by XRD peaks showing a layered perovskite. In addition to a longer PL lifetime (46.2 to 405 ns) indicating suppressed non‐radiative recombination paths, power‐dependent steady‐state PL measurements indicated a lower trap‐state density for OAI‐based perovskite films (0.4 × 10^17^ cm^−3^) compared to control films (2.3 × 10^17^ cm^−3^). By reducing the trap‐state densities and filling up the vacancies, the efficiency of OAI‐based devices was improved from 14.17% to 15.19%. More importantly, an unsealed 2D/3D device retained 86% of its initial efficiency under 50% relative humidity and dark conditions over a period of ≈100 h.

**Figure 6:**
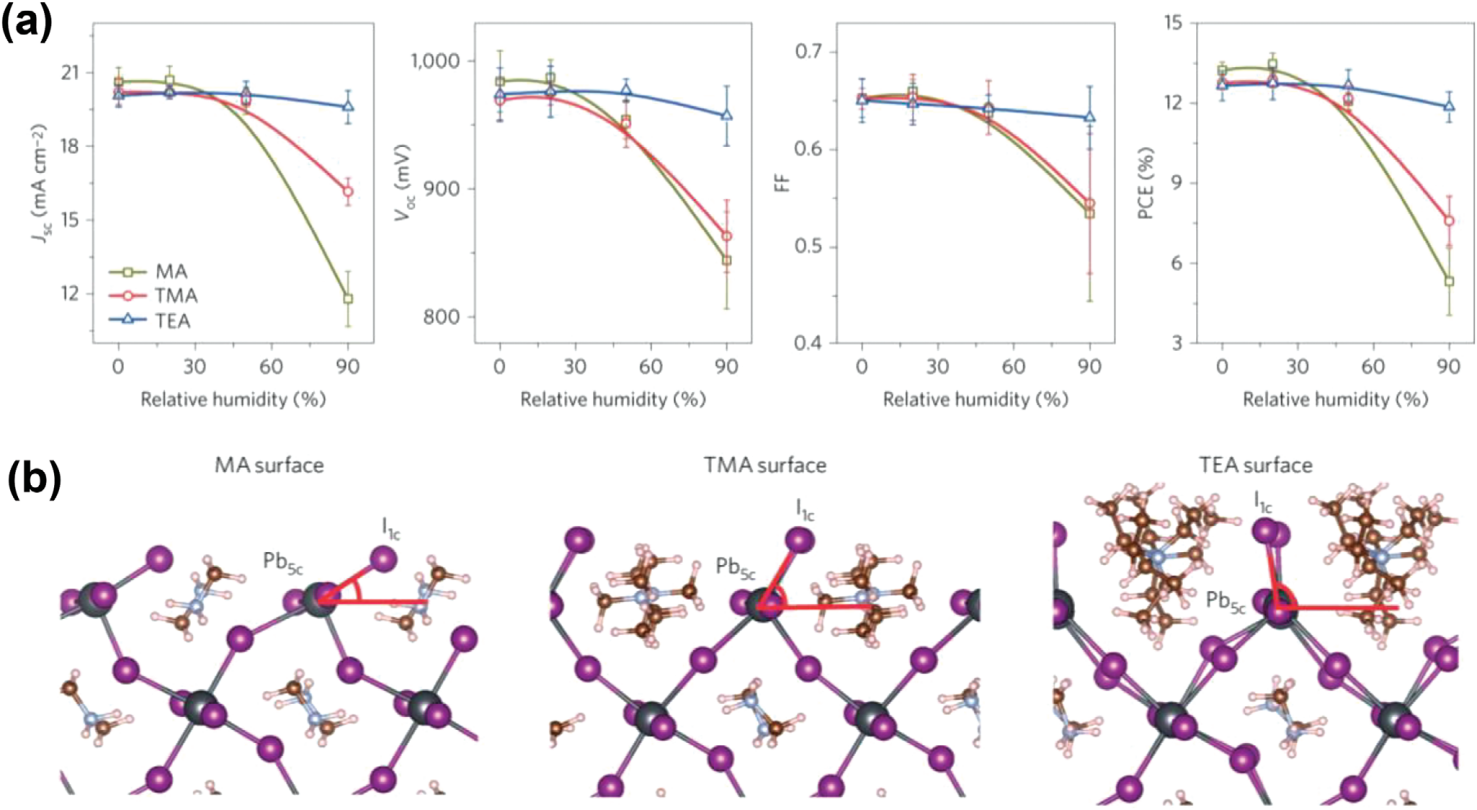
a) Photovoltaic performance of PSCs fabricated after the perovskite layers are exposed to different humidity levels for 24 h. Error bars represent the standard deviation of the measured parameters (minimum five devices). All *J–V* measurements were operated under AM 1.5 G irradiation at a reverse scan rate of 0.15 V s^−1^. b) Side views of the optimized geometries of the (100) surfaces of MA, TMA, and TEA samples. Reproduced with permission.^[^ ^62^ ^]^ Copyright 2020, Springer Nature.

Molecular passivators with large organic substituents normally introduce charge extraction barriers, thus compromising the photovoltaic performance of the corresponding PSC devices, although better protection of the perovskite is anticipated simultaneously. Yang et al. assembled various hydrophobic ammonium cations, varying from relatively small tetra‐methyl ammonium (TMA), tetra‐ethyl ammonium (TEA^+^), and tetra‐butyl ammonium (TBA^+^), to large ammonium cations such as tetra‐hexyl ammonium (THA^+^) and cetyl trimethyl ammonium (CTA^+^), on the MAPbI_3_ perovskite surface to inhibit degradation when exposed to humid conditions.^[^ ^62^ ^]^ The PSC employing a TEA^+^ passivation layer achieved a *J*_SC_ of 19.60 mA cm^−2^, a *V*_OC_ of 990 mV, an FF of 0.67, and a PCE of 12.89%, whereas the typical MA device yielded a PCE of 13.73% under the same conditions (**Figure** **6**a). Despite this decrease in efficiency, perovskite films with a hydrophobic TEA layer were less sensitive to humidity and retained their average parameters even under 90% humidity over 30 days. The ammonium compound bearing the longest alkyl substituent, CTA^+^, can also effectively protect the perovskite against ambient moisture even though it did not improve the initial efficiency. For instance, the water contact angle of pristine MAPbI_3_ films were decreased from 51° to 33° within 1 min, while that of CTA treated film remains in the low wettability range between 90° and 180° over a period of 5 min. The role of the water‐resisting layer was further investigated by first‐principles calculations taking into consideration van der Waals interactions, which confirmed that steric effects caused by bulky hydrophobic ammonium cations and change of surface Pb_5c_—I_1c_ bonds can effectively hinder the adsorption of water on reactive Pb_5c_ sites (Figure 6b).

**Figure 7:**
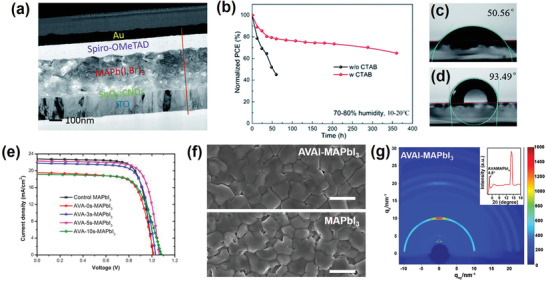
a) Cross‐sectional TEM image of the perovskite cell (treated with CTABr). b) Humidity stability of the devices tested at 10–20°C under 75 ± 5% relative humidity (RH) without any encapsulation. The water contact angle for c) the MAPbI_3_ film and d) the MAPbI_3_ film treated with CTABr. a–d) Reproduced with permission.^[^ ^63^ ^]^ Copyright 2020, Royal Society of Chemistry. e) *J–V* results of the champion cells with different perovskite light absorbers under simulated AM 1.5 radiation. Reproduced with permission.^[^ ^64^ ^]^ Copyright 2020, Wiley‐VCH. f) SEM images of MAPbI_3_ and AVAI‐MAPbI_3_ films. Scale bars, 1 µm. g) GIWAXS data from AVAI‐MAPbI_3_ films. The inset shows the integrated intensity profile for AVAI‐MAPbI_3_ films. f,g) Reproduced with permission.^[^ ^65^ ^]^ Copyright 2020, American Chemical Society.

Wang et al.^[^ ^63^ ^]^ reported a better photovoltaic performance and improved moisture stability for cetyl trimethyl ammonium bromide (CTABr) passivated MAPbI_3_ (**Figure** **7**a). The authors claimed that the ammonium group in CTA^+^ can anchor to the surface and grain boundaries of the perovskite film. The uniform distribution of Br^−^ in CTABr on the surface of the perovskite film was demonstrated using EDX mapping. A PCE of 18.95% with a steady‐state output PCE of 18.11% was achieved after optimizing the concentration of CTABr solution. The stability of the un‐encapsulated devices was tested under 75 ± 5% relative humidity for 360 h (Figure 7b). The CTABr treated device showed better moisture stability and maintained over 65% of its initial PCE, while a sharp decrease in PCE was observed for the pristine device over the first 50 h. This was further supported by the increase in water contact angle from 50.56° for the prinstine perovskite surface, to 93.49° for the perovskite material with CTABr treatment (Figure 7c,d).

Apart from the size effect, aliphatic ammonium halides bearing multiple functionalities have also been studied, aiming to involve additional functionalities and interactions to improve the photovoltaic performance of the prepared PSCs. For instance, Leong et al. utilized 5‐ammoniumvaleric acid iodide, (HOOC(CH_2_)_4_NH_3_I, AVAI), to passivate the defects of 3D MAPbI_3_ perovskite.^[^ ^64^ ^]^ Spin‐casting of the AVAI solution resulted in the formation of a 2D overlayer by reacting with residual PbI_2_, possibly forming a (AVA)_2_PbI_4_ structure at the surface. The presence of the amine and carboxylic acid functional groups in AVAI which can interact with the 3D perovskite through hydrogen‐to‐halogen bonding, lead to lower charge recombination at the corresponding interface as a result of the better contact. The best efficiency of 18.0% was obtained for the AVA‐5s‐MAPbI_3_ (flash‐annealing for 5 s) hybrid device with enhanced stability in both inert (90% of its original PCE after 32 d) and ambient conditions (72% of its original PCE after 20 d), indicating a considerable reduction in nonradiative recombination (Figure 7e).

The role of the AVAI‐based low‐dimensional perovskite on the stability was recently also investigated by Zhao et al. in a typical planar device configuration of FTO/c‐TiO_2_/SnO_2_/perovskite/spiro‐OMeTAD/Ag.^[^ ^65^ ^]^ AVAI treatment of their perovskite films formed a smooth and compact surface morphology associated with significantly reduced pinholes and cracks on the MAPbI_3_ surface (Figure 7f). Despite previous reports assuming the formation of a typical 2D perovskite with the formula AVA_2_PbI_4_ as the main reason for enhanced stability, they showed the formation of AVAMAPbI_4_ mixed‐cation 2D perovskite on the surface of MAPbI_3_ (Figure 7g). The fundamental differences in crystal quality, absorption ability, and surface morphology between AVAMAPbI_4_ and AVA_2_PbI_4_ perovskites were further discussed in this study. The 2D perovskite AVAMAPbI_4_ not only enhanced the thermal and moisture stability of the pristine 3D perovskites but also reduced the amount of undesired charge–carrier recombination, yielding a champion PCE of 20.05%.

Very recently, our group developed a passivation strategy based on a mixture of ammonium salts, CF_3_(CF_2_)_2_CH_2_NH_3_I (HBAI), and a formamidinium halide, FAX (where X is I, Br, or Cl).^[^ ^66^ ^]^ This mixture was applied onto a Cs_0.05_MA_0.1_FA_0.85_PbI_2.9_Br_0.1_ 3D perovskite layer and resulted in the formation of a passivation layer with interacting HBAI and FAX molecules leading to a significant reduction of the amount of nonradiative recombination. The influence of post‐treatment with HBAI·FAX on surface recombination was further confirmed by steady‐state PL and electroluminescence (EL) spectra. An efficiency of 22.78% was obtained in the presence of HBAI·FABr passivation layer while the control device showed a PCE of only 20.1%. In order to understand the improvements with regard to energy level alignment at the interface, we calculated the ionization potentials of perovskites with and without post‐treatment and found that HBAI molecules can up‐shift their valence band to more positive potentials.

### 2.2 Cyclic Aliphatic Organic Substituents

**Figure 8:**
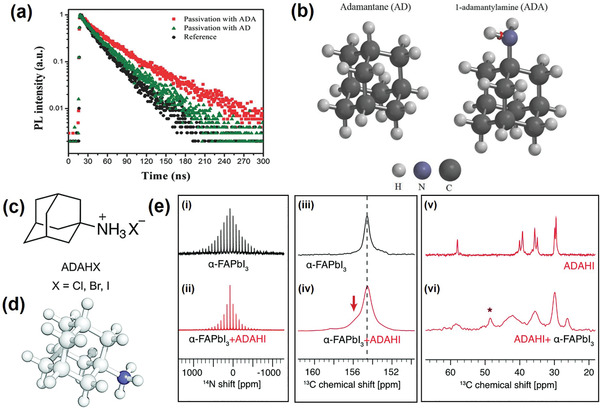
a) TRPL spectra of perovskite films before and after passivation by AD and ADA molecules with optimum concentration of 1.5 mg mL^−1^ in CB using the SC method. b) Chemical structure of adamantane (AD) and 1‐adamantylamine (ADA) used for passivation of perovskite film. a,b) Reproduced with permission.^[^ ^67^ ^]^ Copyright 2020, Wiley‐VCH. c) Chemical structure of ADAHX. d) Optimized geometry (DFT B3LYP/6‐31G(d)) of ADAH^+^. e) ^14^N solid‐state MAS NMR spectra at 21.1 T, 298 K, and 5 kHz MAS of i) *α*‐FAPbI_3_ and ii) *α*‐FAPbI_3_ treated with ADAHI. ^13^C CP solid‐state MAS NMR spectra at 21.1 T, 100 K, and 12 kHz MAS of iii) *α*‐FAPbI_3_ and iv) *α*‐FAPbI_3_ treated with ADAHI (the FA spectral region), as well as v) neat ADAHI powder and vi) *α*‐FAPbI_3_ treated with ADAHI (the ADAHI spectral region). The red arrow indicates a new FA environment present after treatment. The asterisk indicates a spinning sideband of the FA signal. *α*‐FAPbI_3_ was prepared mechanochemically and the treated samples contain 7 mol% of ADAHI. c–e) Reproduced with permission.^[^ ^68^ ^]^ Copyright 2020, Elsevier.

A cyclic aliphatic compound contains at least one all‐carbon ring which may be either saturated or unsaturated but does not have aromatic character. Adamantane is a commonly used polycyclic aliphatic compound with considerable application in pharmaceutical research. Tavakoli et al. introduced adamantane (AD) and 1‐adamantylamine (ADA) as molecular modulators to passivate the electronic defects at the perovskite‐HTL interface.^[^ ^67^ ^]^ As a result of the reduction of surface states acting as sites for nonradiative charge carrier recombination, TRPL measurements indicated significantly longer lifetimes for perovskite films passivated by AD (34.6 ns) and ADA (68.4 ns) compared to the pristine films (14.3 ns) (**Figure** **8**a). ADA also led to an increase of the *V*_OC_ by 60 mV to 1.17 V enabling a stabilized PCE of 21.3%. Importantly, perovskite devices treated with ADA and AD retained over 96% and 92%, respectively, of their initial efficiency after 300 h under continuous light illumination due to the unique films they formed and the strongly hydrophobic properties of the adamantane moiety. The results demonstrated that ADA is slightly more effective in passivating the surface states of perovskite films as compared to AD. This difference was ascribed to a stronger surface attachment of ADA by virtue of the amine group that can anchor to the perovskite surface by replacing an A‐cation or by filling an A‐cation vacancy (Figure 8b).

In another study reported by Tavakoli et al., an effective approach to mitigate the nonradiative recombination of charge carriers at the perovskite/HTL interface by adding protonated 1‐adamantylammonium halide derivatives (ADAHX, X = Cl^−^, Br^−^, I^−^) as molecular modulators into the HTL solution was described (Figure 8c,d).^[^ ^68^ ^]^ In addition to the effects of the ADAHX cation related to eliminating undesirable defects and increasing hydrophobicity, the impact of halide counterions on suppressing halide defects was also investigated. The interaction of ADAHI with the perovskite surface was studied by solid‐state NMR spectroscopy (Figure 8e). Specifically, the ^14^N magic angle spinning (MAS) spectrum of an ADAHI treated sample showed an intrinsic crystallographic symmetry of the *α*‐FAPbI_3_ phase during growth. Moreover, the identical shift in the central peak of ^14^N MAS spectra for both the reference and the treated sample confirmed the interaction of ADAHI with the surface of the perovskite phase in accordance with its large size. Analysis of the photovoltaic properties of finished devices showed that ADAHI gives the highest increase in *V*_OC_, while *J*_SC_ and FF are comparable for all halide derivatives. This effect was explained on the basis of ADAHI as a source of supplemental iodide, whose likely role is to suppress iodide vacancies associated with uncoordinated Pb^2+^ ions on the perovskite surface. Long‐term stability tests were conducted keeping devices in a nitrogen atmosphere under constant illumination and MPP tracking. Remarkably, the device employing ADAHI showed no significant change after 500 h while the reference device lost 18% of its initial efficiency.

**Figure 9:**
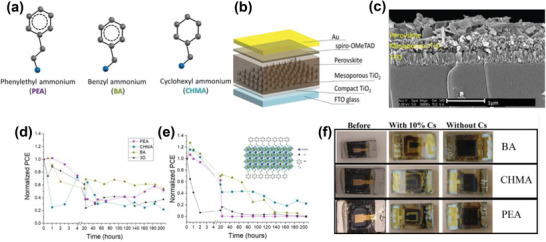
a) Schematic structure of the different barriers (R‐NH_3_): phenethylammonium iodide (PEA), benzylammonium iodide (BA), and cyclohexylammonium iodide (CHMA). b) Solar cell architecture used in this study. c) HR‐SEM cross section of the solar cell with the 2D/3D (*n* = 40) perovskite, where the CHMA is the barrier molecule. d) Stability measurements of solar cells based on 2D/3D perovskites (*n* = 40) of the different barriers and of the 3D perovskite under 1 sun illumination, 30−50% humidity, and for 205 h. e) Stability measurements of solar cells based on 2D/3D mixed cation perovskites (*n* = 40) of the different barriers and of the 3D mixed cation perovskite under 1 sun illumination, 30−50% humidity, and for 205 h. Inset shows the schematic illustration of distorted layered mixed cation MA + Cs perovskite with CHMA as a spacer. f) Photo of the solar cell before the stability measurements (top). Photo of the solar cell based on a mixed cation (Cs + MA) and the corresponding spacer after the stability measurements (middle). Photo of the solar cell based on the corresponding spacer after the stability measurements (bottom). Reproduced with permission.^[^ ^69^ ^]^ Copyright 2020, American Chemical Society.

As another example of a cyclic aliphatic substituent, cyclohexylammonium iodide (CHMA) was employed between perovskite and HTL in a 2D/3D configuration (**Figure** **9**a–c).^[^ ^69^ ^]^ The effect of Cs^+^ cation on the stability and performance of devices was also investigated. The introduction of Cs^+^ cation increased the PCE of both control (from 11.86% to 14.61%) and 2D/3D devices (from 11.54% to 12.58%). However, both 3D and 2D/3D devices displayed relatively poor stability compared to that of Cs‐free devices under one sun illumination at 50–60 °C (Figure 9d–f). This could be tentatively ascribed to the strain in the perovskite structure caused by the presence of two cations with different ionic radii. However, the cells employing CHMA exhibited better stability even in the case of mixed cations. Organic molecular spacers featuring aromatic rings in their backbone, namely phenethylammonium iodide (PEA) and benzylammonium iodide (BA), were also investigated in the same study for comparison. Regarding efficiency, the spacer molecules did not appreciably affect the device performance; however, the stability of the cells was impacted by the presence of these organic molecular spacers. Compared to the cyclic aliphatic spacer, these aromatic spacers lead to improved device stabilities.

### 2.3 Aromatic Organic Substituents

The second family of organic surface treatment agents contains aromatic moieties, making use of their spacer effect and electronic effect simultaneously. As the simplest derivatives of aromatic ammonium iodide, phenylethylammonium iodide (PEAI) and benzylammonium iodide (BAI) are the most widely studied and used aromatic ammonium halide passivation materials.^[^ ^49^ ^]^ It is worth mentioning that the aliphatic spacer between the aromatic ring and ammonium halide is considered essential due to the *π*‐electron delocalization effect of the ammonium halide. Other organic aromatic substituents include thiophene and naphthalene. Due to the size limitation, the aromatic substituents are generally very small *π*‐conjugated molecules, such as the above‐mentioned phenyl, thiophene and naphthalene, whose bandgaps are normally larger than 3.0 eV. As a result, properties other than electronic effects are studied, such as spacer and hydrophobicity effects. The spacer effect is similar to that of aliphatic ammonium halide passivators. However, the phenyl ring simplifies chemical modification.

**Figure 10:**
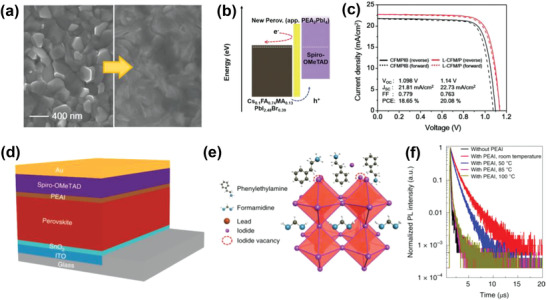
a) Top‐view SEM images of the CFMPIB film (left) and the L‐CFM/P film (right). b) Energy band diagram of the L‐CFM/P device and description of how the 2D perovskite capping layer improved the PCE. c) *J–V* curves and hysteresis of PSCs at a scan rate of 25 mV s^−1^. a–c) Reproduced with permission.^[^ ^70^ ^]^ Copyright 2020, Elsevier. d) The device structure adopted in this study. PEAI is used for post‐treatment of the perovskite surface. e) Possible passivation mechanism of the PEAI layer for the perovskite film. f) TRPL of the perovskite films with PEAI treatment under different conditions. d–f) Reproduced with permission.^[^ ^46^ ^]^ Copyright 2020, Springer Nature.

An innovative approach to controlling the surface growth of a 2D perovskite film on top of a 3D perovskite was designed by using dynamic spin coating of a PEAI‐isopropanol solution, followed by annealing at 100 °C.^[^ ^70^ ^]^ The employment of spin coating in dynamic mode reduced the impact of the isopropanol solution with regard to permeating into the 3D perovskite film and led to the formation of a uniformly thin and distinct PEAI layer (**Figure** **10**a). Despite there being no distinct difference in surface morphology, the root mean square roughness value decreased from 25.01 to 18.76 nm after deposition of PEAI. Thanks to the favorable energy alignment promoting effective hole transfer and electron blocking within the stack (Figure 10b), the devices achieved a maximum PCE of 20.7% (Figure 10c) and retained 90% of their PCE after 800 h of maximum power point tracking under full illumination at a temperature of 50 °C.

One of the highest certified efficiencies of any single‐junction perovskite device (23.32%) was obtained by post‐treatment of FA_1−_ *_x_*MA*_x_*PbI_3_ perovskite surface with phenethylammonium iodide (PEAI) (Figure 10d,e).^[^ ^46^ ^]^ A *V*_OC_ as high as 1.18 V was achieved at the absorption threshold of 1.53 eV, corresponding to 94.4% of the Shockley–Queisser limit (1.25 V). The presence of PEAI on the perovskite surface was revealed by XPS measurements showing a peak at 292.0 eV consistent with *π*–*π* bonding in the phenyl functional group of the PEA^+^ cation. The obvious increase in PL intensity as well as significant increase in carrier lifetime from 0.3 µs to more than 2 µs suggested a considerable suppression of nonradiative recombination defects (Figure 10f). The authors also demonstrated that when exposed to thermal stress by heating to 85 °C, the PCE of the PEAI‐treated cell decreased by ≈10% at the beginning and then remained almost constant up to 500 h of heating.

**Figure 11:**
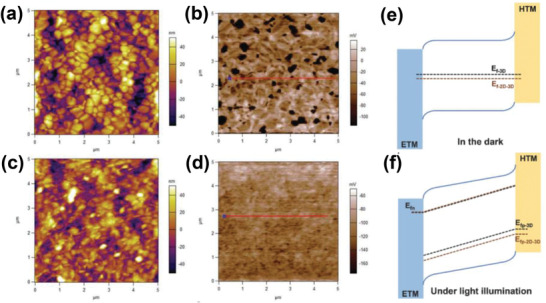
a,b) AFM image and the corresponding CPD distribution image of the 3D perovskite film. c,d) AFM image and the corresponding CPD distribution image of the 2D‐3D perovskite film. e,f) Schematic illustration of the energy band alignment regarding Fermi level splitting in the dark and under light illumination. Reproduced with permission.^[^ ^71^ ^]^ Copyright 2020, Wiley‐VCH.

Chen et al. rationally designed a 2D/3D perovskite stacking‐layered structure by forming a PEA_2_PbI_4_ capping layer on top of a Cs_0.05_(FA_0.83_MA_0.17_)_0.95_Pb(I_0.83_Br_0.17_)_3_ film.^[^ ^71^ ^]^ A 60 mV increase in *V*_OC_ leading to a PCE of 18.51% was achieved when treating the 3D perovskite with a 1 mg mL^−1^ solution of PEAI dissolved in isopropanol due to the larger Fermi level splitting in the 2D/3D perovskite film. The mechanism behind the *V*_OC_ enhancement was further revealed by KPFM results showing a downward shift of the Fermi level comparable to the observed increase in *V*_OC_ (**Figure** **11**a–f). The hydrophobic backbone of the organic spacer also increased device stability through interface passivation preventing water ingress. In addition, a significant improvement in thermal stability was reported for devices following treatment with a 1 mg mL^−^1 PEAI solution, maintaining around 75% of their original PCE at 60 °C for 100 h.

Docampo used the PEA^+^ cation to fabricate a layered perovskite structure using a solution‐based cation infiltration process.^[^ ^47^ ^]^ Preferential orientation of the layered perovskite was revealed by GIWAXS experiments. The layered perovskite was oriented parallel to the substrate which was tentatively explained with a reorganization of the MAPI_3_ top layer upon spin‐coating a PEAI:MAI solution, which resulted in a fast deintercalation of MAI and intercalation of PEAI into the perovskite structure. PEA^+^ cations were found to create extended organic sheets because of steric effects, *π*‐stacking of the phenyl‐rings, and hydrogen‐bonding interactions of the NH_3_^+^ group with the neighboring layer of [PbI_6_] octahedra. This further indicated that organic cations featuring large hydrophobic backbones can act as templates for the anisotropic growth of hybrid perovskite films with a strongly preferred parallel crystal orientation, confining layers of inorganic [PbI_6_] octahedra sandwiched between two organic layers. As a result, heterojunction cells reached a PCE of up to 16.84% due to significant increases in *V*_OC_ and FF.

Despite an enhanced performance and prolonged stability, the interfacial charge transport/recombination mechanisms at the 2D/3D heterojunction are still not totally clear and require deeper understanding. For instance, the ligand‐chemistry‐dependent nature of the 2D/3D heterojunction and possible effects on charge transport and photovoltaic performance, is not yet fully understood. As far as we know, ligand chemistry is crucial for the orientation of 2D perovskites,^[^ ^72^ ^]^ which is expected to play an important role in charge transport at the 2D/3D interface. Niu et al. reported a systematic study aimed at understanding how interfacial engineering using fluorinated spacer ligands or compositional tuning influences the charge transport properties and final photovoltaic performance of FAPbI_3_‐based 2D/3D PSCs.^[^ ^73^ ^]^ Phase‐pure quantum wells were observed for the aliphatic substituents BA and 4,4,4‐trifluorobutylammonium (FBA), while phase impurity of quantum wells was obtained for the aromatic substituents PEA and 4‐fluorophenylethylammonium (FPEA) which could be improved by composition engineering of used substituents. FA:PEA (1:1)‐based films exhibited slightly faster charge transport dynamics compared to PEA‐, FPEA‐, BA‐, and FBA‐based films whose rates were comparable. This indicates a more effective passivation leading to reduced losses from nonradiative recombination. Fluorination of spacers thereby showed a negligible impact on charge transport dynamics. The highest PCEs of 19.50%, 19.84%, and 21.15% were achieved for the devices employing BA‐, PEA‐, and FA:PEA (1:1), respectively, mainly due to a considerable improvement in the *V*_OC_ (1.14 vs 1.05 V) compared to pristine devices. The improvement in *V*_OC_ was explained by a combination of built‐in band alignment and suppressed nonradiative charge recombination. Moreover, the 2D/3D devices displayed significantly improved stability under ambient conditions compared to the control devices, particularly for those featuring more hydrophobic films. More specifically, the FPEA‐based device maintained 84% of its initial PCE after exposure to ambient conditions for 60 days, higher than the PEA:FA (1:1)‐based one (52%) despite its lower PCE.

In a related study, aromatic ammonium cations such as BA^+^, PEA^+^, and FPEA^+^ were introduced to stabilize the metastable FAPbI_3_ phase synthesized at room temperature without cation or anion alloying.^[^ ^74^ ^]^ Characterization by XPS revealed that aromatic ammonium cations bind to the surface of the perovskite structure. According to calculations, this leads to a reduction of the surface energy, which is therefore suggested to play a dominant role in stabilizing the metastable FAPbI_3_ phase. High‐performance solar cells could be fabricated using this directly synthesized, stable, phase‐pure FAPbI_3_ with a lower bandgap.

Yang et.al successfully introduced a new organic compound, 1‐(ammonium acetyl)pyrene (PEY) as the organic spacer for preparing a new 2D/3D heterostructure with planar device architecture.^[^ ^75^ ^]^ Such devices were fabricated under ambient conditions with ≈60% relative humidity at room temperature. Due to the pyrene functional group with high humidity resistance and strong absorption in the ultraviolet region, the 2D/3D perovskite film exhibited nearly no degradation after more than six months storage at around 60% relative humidity, whereas MAPbI_3_‐based control devices quickly degraded over the course of two weeks. Moreover, a high ultraviolet stability was found with nearly no degradation occurring during UV‐ozone treatment for 1 h. The champion PSC employing (PEY_2_PbI_4_)_0.02_MAPbI_3_ reached a PCE of 14.7% with nearly no hysteresis.

Very recently, the Nazeeruddin group reported a slow evolution of the performance of 2D/3D PSCs employing a new family of thiophene alkylammonium‐based organic cations as building blocks for layered 2D perovskites on top of 3D perovskite.^[^ ^76^ ^]^ They revealed that in the case of 2‐thiophenemethylammonium iodide (2‐TMAI) and 3‐thiophenemethylammonium iodide (3‐TMAI), the formed 2D/3D interfaces are dynamic in nature upon aging or exposure to thermal stress, thereby transforming into quasi‐2D (or mixed) phases. The authors monitored this structural change over months, combining solar cell operation with structural and optical characterization of thin films of aged samples and following thermal stress. They found that 2D perovskites of 2‐TMAI and 3‐TMAI can embed small MA or FA ions migrating from the 3D bulk underneath immobilizing them into new quasi‐2D structures. Therefore, deposition of a 2D layer with different thiophene alkylammonium iodide cations was able to protect the 3D layer from degradation in ambient air improving the device performance obtainable after aging in the dark, but not leading to stable device operation under illumination. On the other hand, they obtained a more robust 2D layer when using 2‐thiopheneethylammonium iodide (2‐TEAI) which was stable upon aging or thermal stress. This prevented the aforementioned structural change and led to a considerable improvement of device stability, retaining 90% of the initial PCE under continuous illumination over 1000 h.

The role of thermal stress on 2D perovskites is another key aspect, which is also a recognized cause of perovskite device degradation. This calls for an in depth understanding of the interface behavior upon heating, which is crucial to assess device stability.^[^ ^77^ ^]^ Another study from the same group followed the slow dynamic structural variation of the 2D perovskite layer at a 2D/3D interface upon being exposed to thermal stress by performing combined in situ GIWAXS with steady‐state and time‐resolved PL measurements in two systems using 2‐TMAI and PEAI, respectively.^[^ ^78^ ^]^ A thin layer (≈60 nm) of 2D perovskite was formed on top of the [(FAPbI_3_)_0.87_(MAPbBr_3_)_0.13_]_0.92_(CsPbI_3_)_0.08_‐based 3D perovskite film by spin coating of the organic salts dissolved in isopropanol. They monitored the structural evolution of the interface upon exposing the samples to a thermal cycle, simulating real life operating conditions. They revealed a dynamic transformation of the 2D into a mixed 2D/3D phase, a process which could protect the 3D perovskite from degradation observed with 2D‐free devices.

**Figure 12:**
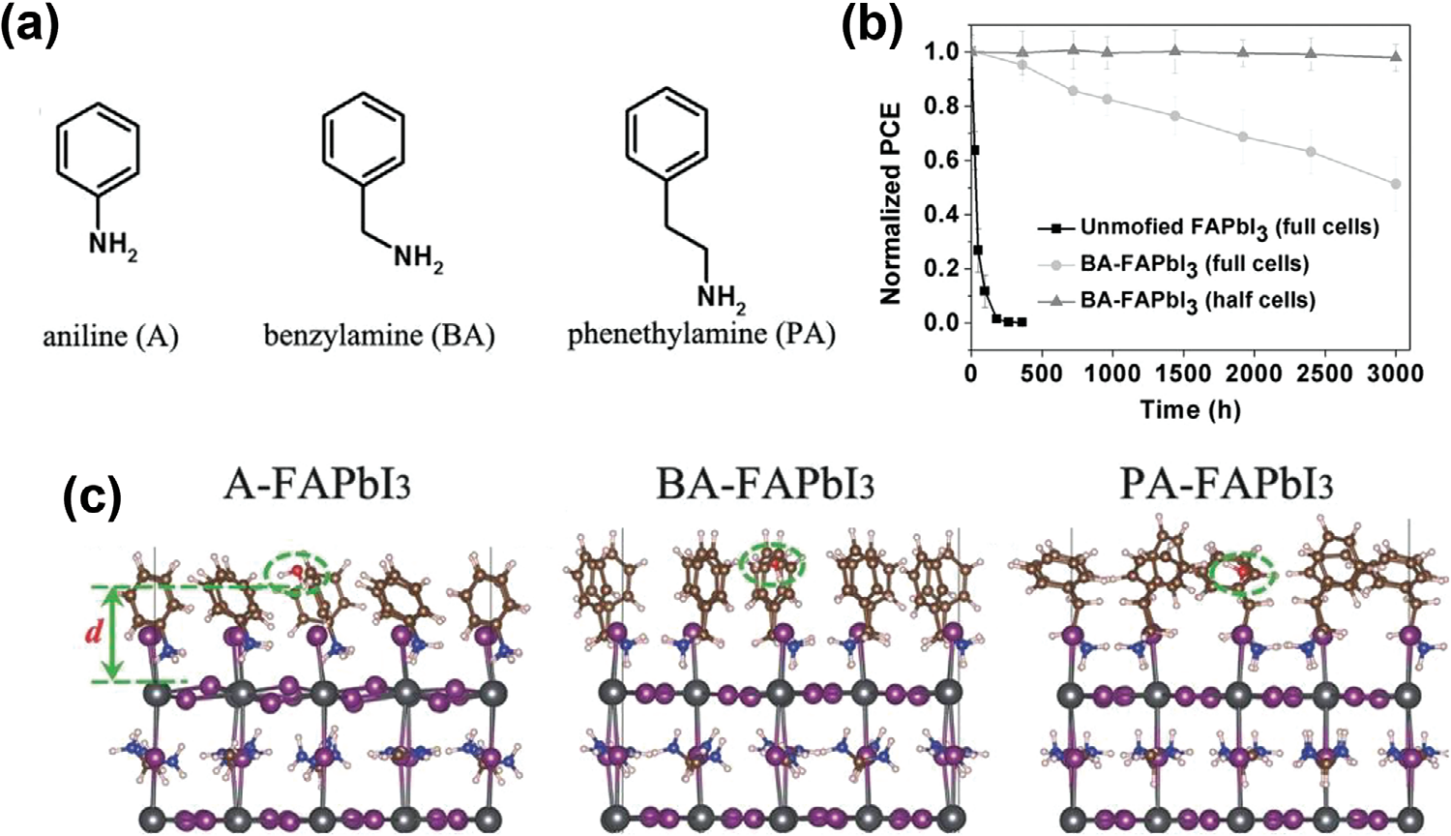
a) The chemical structures of aniline, benzylamine, and phenethylamine. b) Moisture stability of unmodified FAPbI_3_ (black line) and BA‐FAPbI_3_ (red and blue lines) devices under air exposure (50 ± 5 RH%). “Half cells” means that only the BA‐FAPbI_3_ films on the TiO_2_/FTO substrates were exposed to air and that the spiro‐OMeTAD and Au layers were deposited onto the films before the *J–V* measurement. c) DFT simulation of one water molecule adsorption on the A‐FAPbI_3_, BA‐FAPbI_3_, and PA‐FAPbI_3_ surface. Reproduced with permission.^[^ ^49^ ^]^ Copyright 2020, Wiley‐VCH.

The resistance to water of the passivated perovskite layers is exceedingly sensitive to the steric arrangement of the passivator molecules. To examine this phenomenon more closely, Zhao et al. designed a study based on benzene as an example of a sterically demanding hydrophobic group, and compared the role of structurally similar benzene containing amines, namely aniline (A), benzylamine (BA), and phenethylamine (PA), on the passivation of FAPbI_3_ perovskite (**Figure** **12**a).^[^ ^49^ ^]^ Comparing these three molecules, only BA passivated FAPbI_3_ remained unchanged for >2900 h of exposure to air at a relative humidity of 50 ± 5% (Figure 12b), despite the similarity of their chemical structures. This result was explained on the basis of the steric arrangement of the amine molecules which was studied by DFT. According to these results, the BA molecules were packed in an almost perpendicular orientation to the perovskite surface, while the others oriented in a relatively irregular fashion. Looking into water adsorption onto these passivated surfaces, larger distances between water molecules and the surface of the Pb–I lattice were found in BA‐FAPbI_3_ compared to the A and PA systems (Figure 12c). In addition, the efficiency of PSCs based on BA reached 19.2% with a *V*_OC_ of 1.12 V, representing the lowest loss‐in‐potential for a perovskite solar cell. This high *V*_OC_ was due to a reduction of recombination sites following treatment with BA, as confirmed by PL and transient photovoltage (TPV) measurements.

**Figure 13:**
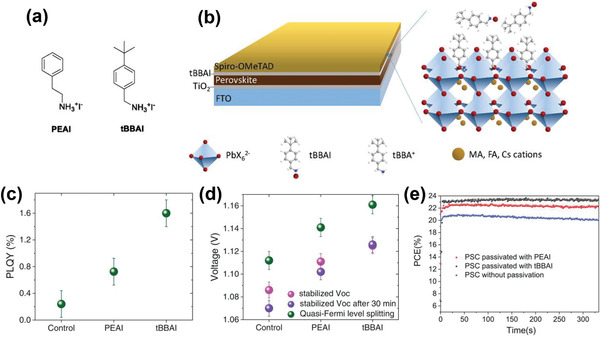
a) Chemical structures of PEAI and *t*BBAI. b) Structures of a *t*BBAI‐passivated PSC. c) PLQY for the layer structure glass/FTO/c‐TiO_2_/mp‐TiO_2_/perovskite/interface layer with HTL. d) Stabilized *V*_OC_ and quasi‐Fermi level splitting *Δ*_EF_/q for the layer structure glass/FTO/compact‐TiO_2_/mesoscopic‐TiO_2_/perovskite/interface layer/HTL. The stabilized *V*_OC_ after 30 min light soaking is also shown. e) MPP tracking of the PSCs within the first 330 s under ambient air. Reproduced with permission.^[^ ^79^ ^]^ Copyright 2020, Wiley‐VCH.

Based on these results, our group introduced a judiciously engineered new passivator 4‐*tert*‐butyl‐benzylammonium iodide (*t*BBAI) (**Figure** **13**a,b).^[^ ^79^ ^]^ Thanks to its bulky *tert*‐butyl group, undesired aggregations, which occurred when using PEAI, were prevented by steric repulsion. Moreover, a rapid charge extraction from the perovskite into the HTL was achieved, while retarding nonradiative charge carrier recombination. The improved photoluminescence quantum yield (PLQY) (Figure 13c) accompanied by larger quasi‐Fermi level splitting in the perovskite films supported that *t*BBAI showed significant passivation, which resulted in a high *V*_OC_ of 1.142 V (Figure 13d). Furthermore, a high PCE of 23.5% (Figure 13e), as well as an outstanding moisture stability due to the hydrophobic *tert*‐butyl group was reported. Specifically, a *t*BBAI passivated device retained over 95% of its initial PCE after 500 h under MPP tracking.

**Figure 14:**
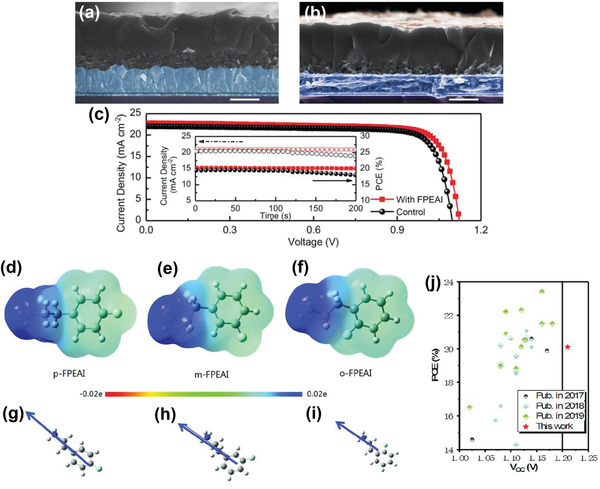
Cross‐sectional SEM images of a) unmodified 3D perovskite) b) (FPEA)_2_PbI_4_ modified perovskite. Scale bar is 400 nm; LSCM images show the emission between 700 and 760 nm (red color for perovskite) and emission between 500 and 550 nm (green color for 2D (FPEA)_2_PbI_4_). Color saturation scales with emission intensity. Excitation laser wavelength at 488 nm. c) *J–V* curves, and (inset) stabilized PCEs with current density at a maximum power point (modified: 0.96 V; control: 0.95 V) of the best performing devices for 200 s. a–c) Reproduced with permission.^[^ ^80^ ^]^ Copyright 2020, Wiley‐VCH. EPS images for d) *p*FPEAI, e) *m*FPEAI, and f) *o*FPEAI. EPS The direction and intensity of molecular dipole moments for g) *p*FPEAI, h) *m*FPEAI, and i) *o*FPEAI as indicated by the length of the arrows. j) Perovskite devices with PCE and *V*_OC_ published in recent years (solid dots represent triple‐cation perovskites; hollow dots represent all other types perovskites. d–j) Reproduced with permission.^[^ ^82^ ^]^ Copyright 2020, Wiley‐VCH.

The effect of fluorine substituted phenyl groups on the moisture tolerance of the resulting materials was examined recently. 2‐(4‐fluorophenyl)ethyl ammonium iodide (FPEAI) was reported to passivate Cs/FA/MA triple‐cation 3D perovskite.^[^ ^80^ ^]^ FPEAI solution prepared in isopropanol (2 mg mL^−1^) was spin‐coated on top of the as‐prepared 3D perovskite and annealed at 100 °C for 5 min to obtain a 2D capping layer. The spatial distribution of the in situ formed 2D perovskite atop the 3D matrix was visualized by using a laser scanning confocal microscope (LSCM) (**Figure** **14**a,b). The LSCM image of pristine 3D perovskite showed exclusive red emission between 700 and 760 nm, whereas concentration‐dependent bright greenish emission was collected between 500 and 550 nm in the presence of 2D perovskite. This capping layer enhanced the inter‐connection between perovskite and HTL, thus significantly lowering charge recombination while improving interfacial hole extraction. The role of 2D perovskites with respect to carrier extraction is undeniably important. In this regard, low‐dielectric‐confined 2D materials might be promising in boosting the performance of hybrid perovskite cells owing to their relatively low exciton‐binding energy.^[^ ^81^ ^]^ Furthermore, high dielectric constants of organic modulators could result in an enhanced carrier mobility associated with the increased relaxation time. As a result of low binding energy and high mobility, such materials are expected to present much better photo‐excited carrier extraction efficiency. This study also showed that the introduction of fluorinated moieties has a considerable effect on the enhancement of hydrophobicity of the 2D capping layer. The best‐performing device employing this novel capping layer yielded a PCE of 20.54% (Figure 14c) and sustained 99% of its initial efficiency after 860 h in the dark with controlled humidity between 10% and 30%.

In order to investigate the effects of the relative position of the fluoro‐substituent (*ortho‐*, *meta‐*, and *para‐*) on the photovoltaic performance and stability of PSCs, Zhou et al. employed three mono‐fluorinated aryl ammonium iodides, namely ((2‐(2‐fluorophenyl)ethylamine iodide (*o*FPEAI), 2‐(3‐fluorophenyl)ethylamine iodide (*m*FPEAI), and 2‐(4‐fluorophenyl)ethylamine iodide (*p*FPEAI)).^[^ ^82^ ^]^ The simulated electrostatic potential surface (EPS) images demonstrated that these three passivating molecules can passivate negatively charged defects at the surface of the used Cs_0.05_FA_0.79_MA_0.16_PbI_2.49_Br_0.51_ perovskite (Figure 14d–f). Moreover, the sequence of *p*FPEAI  >  *m*FPEAI  >  *o*FPEAI in dipole moment was observed, indicating better charge separation, and hole transfer at the perovskite interface (Figure 14g–i). The passivated cells yielded improved PCEs of 20.60%, 20.52%, and 20.37% for *o*FPEAI, *m*FPEAI, and *p*FPEAI, respectively, with a notable increase in *V*_OC_ (Figure 14j). *o*FPEA exhibited the best passivation effect possibly due to the highest energy of formation while *p*FPEA‐treated perovskite demonstrated the best moisture stability associated with the highest hydrophobicity.

In addition to the photovoltaic performance and stability of PSCs, the hysteresis phenomenon is another issue that needs to be solved ahead of their future commercialization. The influence of phenylalkylammonium iodides (C_6_H_5_)(CH_2_)_n_(NH_3_I) featuring alkyl chains of different lengths (*n* = 0, 1, and 2) on the hysteresis was first reported by Yoo et al. using the normal mesoporous structure employing c‐TiO_2_/mp‐TiO_2_/perovskite/spiro‐OMeTAD configuration.^[^ ^83^ ^]^ In this study, the surface of FA_0.9_Cs_0.1_PbI_2.9_Br_0.1_ perovskite was passivated with phenylammonium iodide (PAI, *n* = 0), phenylmethylammonium iodide (PMAI, *n* = 1), and phenylethylammonium iodide (PEAI, *n* = 2). An improvement in PCE (from 16.47% to 17.32% and to 18.09%) with increasing alkyl chain length was achieved and mainly ascribed to the increase in *V*_OC_ from 0.979 V (PAI) to 1.022 V (PMAI) and 1.035 V (PEAI), indicating an effective suppression of charge recombination at the perovskite/spiro‐OMeTAD interface. Additionally, they found that the hysteresis index decreased as the alkyl chain length increased due to the decrease in nonradiative recombination along with an increased carrier lifetime. In other words, the short length of PAI was not sufficient to compensate the trapped charge in the charge accumulation layer. An effect of the solvent used for preparing the phenylalkylammonium solution was also reported in this study, showing that IPA is a more appropriate solvent compared to ethanol or methanol.

**Figure 15:**
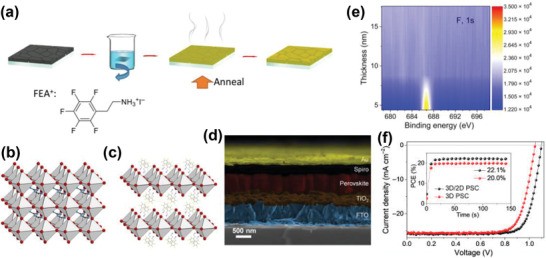
a) Schematic illustration of the 2D treatment of 3D perovskite to form the 3D/2D bilayer perovskite with the structural representation of the FEA^+^ cation and the corresponding optimized geometry [density functional theory (DFT) calculation on B3LYP/6‐31G(d) level of theory]. b) Structures of pure 3D perovskite and c) pure 2D perovskite. d) Cross‐sectional SEM of 3D/2D PSC. e) Fluorine XPS in‐depth profiling of 3D/2D bilayer perovskite. f) *J‒V* curves of a 3D PSC and a 3D/2D PSC, with inset showing MPP tracking. Reproduced with permission under the terms of the CC BY‐NC 4.0 license.^[^ ^7^ ^]^ Copyright 2020, The Authors, some rights reserved, exclusive license American Association for the Advancement of Science.

Very recently, our group exploited this by inserting an ultrahydrophobic pentafluoro‐phenylethylammonium (FEA) lead iodide [(FEA)_2_PbI_4_] 2D perovskite layer between the 3D perovskite and HTL in the configuration of FTO/c‐TiO_2_/mp‐TiO_2_/perovskite/spiro‐OMeTAD/Au (**Figure** **15**a–d).^[^ ^84^ ^]^ XPS depth profiling of fluorine (F) confirmed the presence of a ≈9 nm thick FEA layer (Figure 15e), whereas EDS mapping showed a conformal and uniform distribution of FEA^+^ over the 3D perovskite layer. Highly fluorinated compounds are normally hydrophobic and hardly wetted by water,^[^ ^85^ ^]^ therefore protecting the perovskite film from ambient moisture while the perfluorinated benzene unit can also promote hole extraction while inhibiting interlayer ion migration. The 2D/3D FEA‐based PSCs showed superior stability maintaining 90% of their initial efficiency in ambient conditions with a prevalent humidity of 40% after 1000 h. PCEs as high as 22.2% were achieved for bilayer‐based PSCs while the control cell yielded an efficiency of 20.6% under the same conditions (Figure 15f). The increase in PCE was ascribed to a twofold improvement in the charge–carrier lifetime and faster hole injection into the HTL, implying the suppression of nonradiative carrier recombination at the perovskite/HTL interface in the presence of the passivation layer.

As an interface layer implemented into a photovoltaic device, an electronically active material is preferred as compared to an insulator, since the latter is considered to create a charge extraction barrier. Although conductive organic materials normally employ increased conjugation size in order to lower the HOMO–LUMO gap, and large ammonium halide passivators are difficult to incorporate into perovskite materials due to steric hindrance, research interest has also focused on triphenylamine to design a p‐type semiconductive molecular modulator.

**Figure 16:**
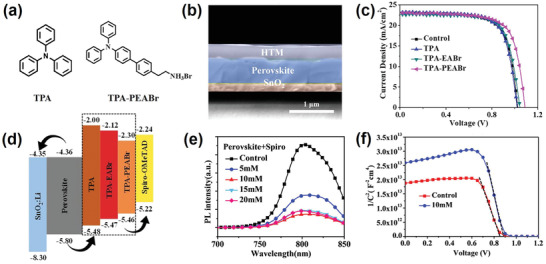
a) Structure of the surface passivation molecules. b) Cross‐sectional SEM image of the full device. c) *J–V* characterization for control and passivated PSCs. The *J–V* curves are measured under standard conditions with a scan rate of 0.1 V s^−1^. d) The band alignment diagram of the PSCs used in this study. e) PL spectra for the 0 to 20 mm TPA‐PEABr‐passivated perovskite/HTM films. f) Mott−Schottky plots of the control and 10 mm TPAPEABr‐passivated PSCs. The data are collected at 5000 Hz. Reproduced with permission.^[^ ^50^ ^]^ Copyright 2020, American Chemical Society.

Recently a small organic molecule, namely *N*‐((4‐(*N*,*N*,*N*‐triphenyl)phenyl)‐ethyl)ammonium bromide (TPA‐PEABr), has been synthesized and employed as a surface passivation molecule in planar device configuration (**Figure** **16**a,b).^[^ ^50^ ^]^ TPA as a charge carrier moiety for spiro‐OMeTAD can facilitate an efficient carrier transfer, whereas alkylammonium‐based PEABr can provide a strong anchoring to the perovskite surface. 10 mm‐TPA‐PEABr passivation enhanced the efficiency of PSCs from 16.69% to 18.15% (Figure 16c). This significantly improved efficiency of passivated devices was ascribed to the effective healing of both negatively and positively charged surface defects and well‐matched energy levels (Figure 16d) in the presence of the TPA‐PEABr buffer layer. The efficient defect passivation reduces the charge trap density and extends the carrier recombination lifetime, which was supported by PL and Mott–Schottky results (Figure 16e–f). The fluorescence quantum yields of the pristine and TPA‐PEABr passivated films were found to be 4.66% and 3.01%, respectively. The relatively low quantum yield of the passivated material can be explained by the carrier transfer between the perovskite and TPA layers.

## 3 Counter Ion Effect

In addition to the cation backbone of alkylammonium salts, the halide counterions also have a significant influence on the performance and stability of passivated perovskite devices by influencing the make‐up of the crystal structure and energetic behavior of the perovskite photoabsorber.^[^ ^87^, ^88^ ^]^ Studied halide counterions normally include chloride (Cl^−^), bromide (Br^−^), and iodide (I^−^). I^−^ ions have demonstrated a promising role in passivation of halide vacancies due to their compatibility with iodide‐rich perovskites. From the previous chapters of this review, it can also be observed that most of the high‐performing passivation systems employ iodide as counterion.^[^ ^54^ ^]^

Due to a reduced orbital overlap between the halide and Pb^2+^ as compared to I^−^, Br^−^ based perovskites normally exhibit a larger bandgap. As a result, ion exchange between Br^−^ containing ammonium halides has proven to be able to increase the photovoltage of the corresponding PSCs by band bending at the interfaces between the perovskite photoabsorber and HTL.^[^ ^53^ ^]^

Although Cl^−^ ions are reported to be able to retard the crystallization rate to obtain larger grains with better morphology, they are less explored as a counterion for passivation agents due to the radius mismatch of Cl^−^ with the 3D perovskite matrix.^[^ ^51^, ^52^ ^]^ The choice of halide counterion therefore needs to be informed by the PSC parameter targeted for improvement. For instance, in order to increase FF and *V*_OC_ by defect passivation, I^−^ is the best choice, while Br^−^ is advantageous for improving *V*_OC_ via band bending.^[^ ^53^, ^59^ ^]^ In addition to band bending, Br^−^ also exhibits the ability to induce Ostwald ripening, thereby decreasing recombination associated charge losses at grain boundaries.^[^ ^89^ ^]^

A systematic study providing a better understanding of the effects of organic ammonium salts with different halogen functional groups was reported by Grancini et al.^[^ ^90^ ^]^ They investigated the effect of 2D/3D perovskite interfaces on the effective reduction of the interfacial energy losses by employing a set of three novel compounds, namely 2‐thiophenemethylammonium halides (2‐TMAX, where X = Cl, Br, I), on a triple‐cation perovskite of [(FAPbI_3_)_0.87_(MAPbBr_3_)_0.13_]_0.92_(CsPbI_3_)_0.08_ composition. Since thiophene ammonium halide salts can easily react with excess PbI_2_ and form 2D structures, the formation of these 2D perovskites was controlled by excess PbI_2_ present in the 3D perovskite. With regard to surface morphology, the formation of the 2‐TMAI and 2‐TMABr‐based 2D layers resulted in smoother surfaces with distinct grain boundaries, while 2‐TMACl led to a distribution of distinct phases with larger and more elongated grains. Furthermore, optical analyses revealed a lower band gap for 2‐TMAI‐treated perovskite films compared to 2‐TMABr and 2‐TMACl. Energetic alignment studies at the 2D/3D interface showed that TMABr and TMAI lead to a beneficial energy level alignment promoting hole extraction by shifting the valence band, while 2‐TMACl exhibited an opposing trend. Differences in the material properties were also reflected in their respective PV results. 2‐TMABr treated devices demonstrated a significant increase in *V*_OC_, delivering a champion PCE of 20.82% with the devices retaining 75% of their initial efficiency under continuous illumination for 1000 h in argon atmosphere without any encapsulation. On the other hand, the observed small decrease in *J*_SC_ was explained on the basis of the increased charge transport barrier induced by the presence of the bulky organic compounds.

## 4 Conclusions and Outlook

PSCs hold much promise for the future of photovoltaics. Despite the stunning progress in terms of efficiency, meeting the high requirements with regard to degradation is still a challenge and major efforts are currently directed toward addressing both intrinsic and environmental degradation mechanisms. One of the ways to overcome these limitations is the investigation of interface engineering using next‐generation functional materials. Thanks to their low‐cost, facile production, and tunable electro‐chemical properties, hybrid perovskites incorporating organic ammonium halides are now emerging as one of the most credible approaches to improving the stability of perovskite devices without sacrificing the spectral features of 3D perovskites. This review summarizes the latest research progress in this direction and provides an overview of recent advancements regarding organic ammonium halides and their key roles in defect‐healing processes, as well as improving the stability of regular type PSCs against detrimental conditions, especially moisture. As discussed in the “Organic Substituent Effect” section, molecular modulators employing different types of organic backbones used for the preparation of ammonium ligands play a substantial role in obtaining high‐efficiency and long‐term stable PSCs. In this context, the choice of organic ammonium cation bearing different substituents results in different interlayer structures, with halide counterions also having a significant impact on the performance and stability of the obtained devices by altering different optoelectronic properties. The effect of the chain length of electrochemically inert aliphatic substituents was also reviewed. Shorter alkyl chains are considered to create lower charge extraction barriers compared to their longer counterparts, however, even cations as small as ethylammonium are not able to be incorporated into the 3D perovskite matrix. In addition, shorter alkyl chains have a reduced ability to create a hydrophobic barrier for the passivated perovskite. On the other hand, longer alkyl chains in molecular passivators provide a more robust protection for the perovskite, however, they come with an increased risk of creating a charge extraction barrier between perovskite and HTL. Aromatic organic substituents have also been widely studied and applied in n–*i*–p‐type devices, with beneficial effects to the photovoltaic performance as well as operational stability of the corresponding PSCs. Although the optical and electronic effects are rarely studied, pioneering studies on semiconducting molecular passivators have already proven the unique advantage of charge–extraction barrier‐free interfacial layers in PSCs.^[^ ^50^ ^]^ Future progress in the field of optically and electronically active molecular passivators are anticipated to be a hot topic in the near future. However, it remains challenging to explicitly define the type and concentration of defect‐states on perovskite films or at perovskite/HTL interfaces. Researchers working in this field need to advance pioneering characterization techniques combined with theoretical guidance which can precisely explore the defects in perovskites. Moreover, developing multi‐functional molecular modulators for a better passivation effect to both grain boundaries and surfaces is considered potentially useful. These approaches require an in‐depth understanding of the properties of passivators and mechanisms of passivation, as well as a thorough grasp of their influence on electronic properties of the passivated perovskites. Scaling up from laboratory‐scale cell fabrication based on chemical approaches to large‐scale industry‐compatible deposition is another crucial point to consider for deployment of this PV technology. Specifically, the deposition of complicated and time‐consuming protective interlayers between perovskite and HTL becomes more critical to avoid the formation of voids, poor coverage, and large thickness variation associated with the organic solvents in standard lithographic processes. In this regard, orthogonal electron beam lithography technique to avoid direct contact of the perovskite with the hexane and chlorobenzene orthogonal solvents causing deterioration of the underlying perovskite films may pave the way for potential breakthroughs in perovskite devices for mass production.^[^ ^91^ ^]^

Although organic ammonium halide molecular modulators have been shown to be able to play a substantial role in improving photovoltaic performance and operational stability by reducing recombination centers and providing humidity resistance, further investigations related to a better understanding of the underlying mechanisms as well as improved molecular designs are still required to reach the commercialization goals of perovskite technology.

## Conflict of Interest

The authors declare no conflict of interest.
